# Structural basis for potency differences between GDF8 and GDF11

**DOI:** 10.1186/s12915-017-0350-1

**Published:** 2017-03-03

**Authors:** Ryan G. Walker, Magdalena Czepnik, Erich J. Goebel, Jason C. McCoy, Ana Vujic, Miook Cho, Juhyun Oh, Senem Aykul, Kelly L. Walton, Gauthier Schang, Daniel J. Bernard, Andrew P. Hinck, Craig A. Harrison, Erik Martinez-Hackert, Amy J. Wagers, Richard T. Lee, Thomas B. Thompson

**Affiliations:** 1 Department of Molecular Genetics, Biochemistry, and Microbiology, University of Cincinnati, Cincinnati, OH 45267 USA; 2 Harvard Stem Cell Institute and Department of Stem Cell and Regenerative Biology, Harvard University, Cambridge, MA 02138 USA; 3 Paul F. Glenn Center for the Biology of Aging, Harvard Medical School, Boston, MA 02115 USA; 4 Department of Biochemistry and Molecular Biology, Michigan State University, East Lansing, MI 48824 USA; 5 Hudson Institute of Medical Research, Clayton, Australia; 6 Department of Pharmacology and Therapeutics, McGill University, Montréal, Quebec Canada; 7 Department of Structural Biology, University of Pittsburgh School of Medicine, Pittsburgh, PA 15260 USA; 8 Department of Physiology, Monash University, Clayton, Australia; 9 University of Cincinnati, 231 Albert Sabin Way ML 0524, Cincinnati, OH 45267 USA

**Keywords:** Ligands, Myostatin, Receptor, Structure, Transforming growth factor β (TGFβ)

## Abstract

**Background:**

Growth/differentiation factor 8 (GDF8) and GDF11 are two highly similar members of the transforming growth factor β (TGFβ) family. While GDF8 has been recognized as a negative regulator of muscle growth and differentiation, there are conflicting studies on the function of GDF11 and whether GDF11 has beneficial effects on age-related dysfunction. To address whether GDF8 and GDF11 are functionally identical, we compared their signaling and structural properties.

**Results:**

Here we show that, despite their high similarity, GDF11 is a more potent activator of SMAD2/3 and signals more effectively through the type I activin-like receptor kinase receptors ALK4/5/7 than GDF8. Resolution of the GDF11:FS288 complex, apo-GDF8, and apo-GDF11 crystal structures reveals unique properties of both ligands, specifically in the type I receptor binding site. Lastly, substitution of GDF11 residues into GDF8 confers enhanced activity to GDF8.

**Conclusions:**

These studies identify distinctive structural features of GDF11 that enhance its potency, relative to GDF8; however, the biological consequences of these differences remain to be determined.

**Electronic supplementary material:**

The online version of this article (doi:10.1186/s12915-017-0350-1) contains supplementary material, which is available to authorized users.

## Background

The transforming growth factor β (TGFβ) superfamily of secreted proteins comprises more than 30 structurally related, yet functionally distinct proteins that play critical roles in embryological tissue development and patterning, wound healing, and adult tissue maintenance (reviewed in [1–5]). The TGFβ superfamily can be divided into three subclasses: TGFβs, bone morphogenetic proteins (BMPs), and activins/inhibins. Growth/differentiation factors 8 and 11 (GDF8/myostatin and GDF11/BMP11, respectively) are two closely related members of the activin/inhibin subclass that share ~90% sequence identity in their mature, C-terminal signaling domains and ~52% identity in their N-terminal prodomains. GDF8 and GDF11 bind to similar receptors [6–8] and extracellular antagonists [6, 9, 10], leading to the assumption that mature GDF8 and GDF11 ligands are functionally indistinguishable. While it is clear that GDF8 and GDF11 share many commonalities, including overlapping or redundant roles in certain biological processes [11–17], there is accumulating evidence that the two ligands may be functionally distinct [13, 18–23].

*Gdf8* is expressed postnatally by skeletal and cardiac muscle and therein negatively regulates skeletal muscle mass by suppressing both the number and size of individual muscle fibers [6, 18, 19, 24]. In contrast, GDF11 appears to act more broadly, regulating anterior/posterior patterning and development of multiple organs/tissues [11, 13]. Many tissues express *Gdf11* postnatally, including the spleen, pancreas, kidney, and skeletal muscle [11, 25–28]. However, determination of GDF11’s exact role in the adult has remained elusive due to the embryonic lethality of *Gdf11* ^*-/-*^ mice [11, 13]. In stark contrast, *Gdf8* ^*-/-*^ mice survive into adulthood and have a profound hypermuscular phenotype, which can be recapitulated in wild-type mice using natural occurring antagonists of GDF8, such as follistatin (FS), follistatin-like 3 (FSTL3), and growth/differentiation factor-associated serum protein 1 (GASP1) [6, 29–33]. Interestingly, *Gdf8* ^*-/-*^ */Gdf11* ^*-/-*^ mice have exaggerated homeotic axial transformations compared to *Gdf11* ^*-/-*^ mice, suggesting that GDF8 and GDF11 have redundant functions in skeletal patterning [13]. However, muscle-specific knockout of *Gdf11* does not result in significant increases in muscle mass and circulating GDF11 does not overcome the hypermuscular phenotype found in *Gdf8* ^*-/-*^ mice, suggesting that GDF8 and GDF11 do not serve redundant roles in regulating skeletal muscle mass [13]. Thus, while it is clear that loss of one ligand compared to the other yields drastically different phenotypes, it has been argued that these differences relate primarily to differential localization of ligand expression and do not reflect differences in ligand signaling.

**Fig. 1:**
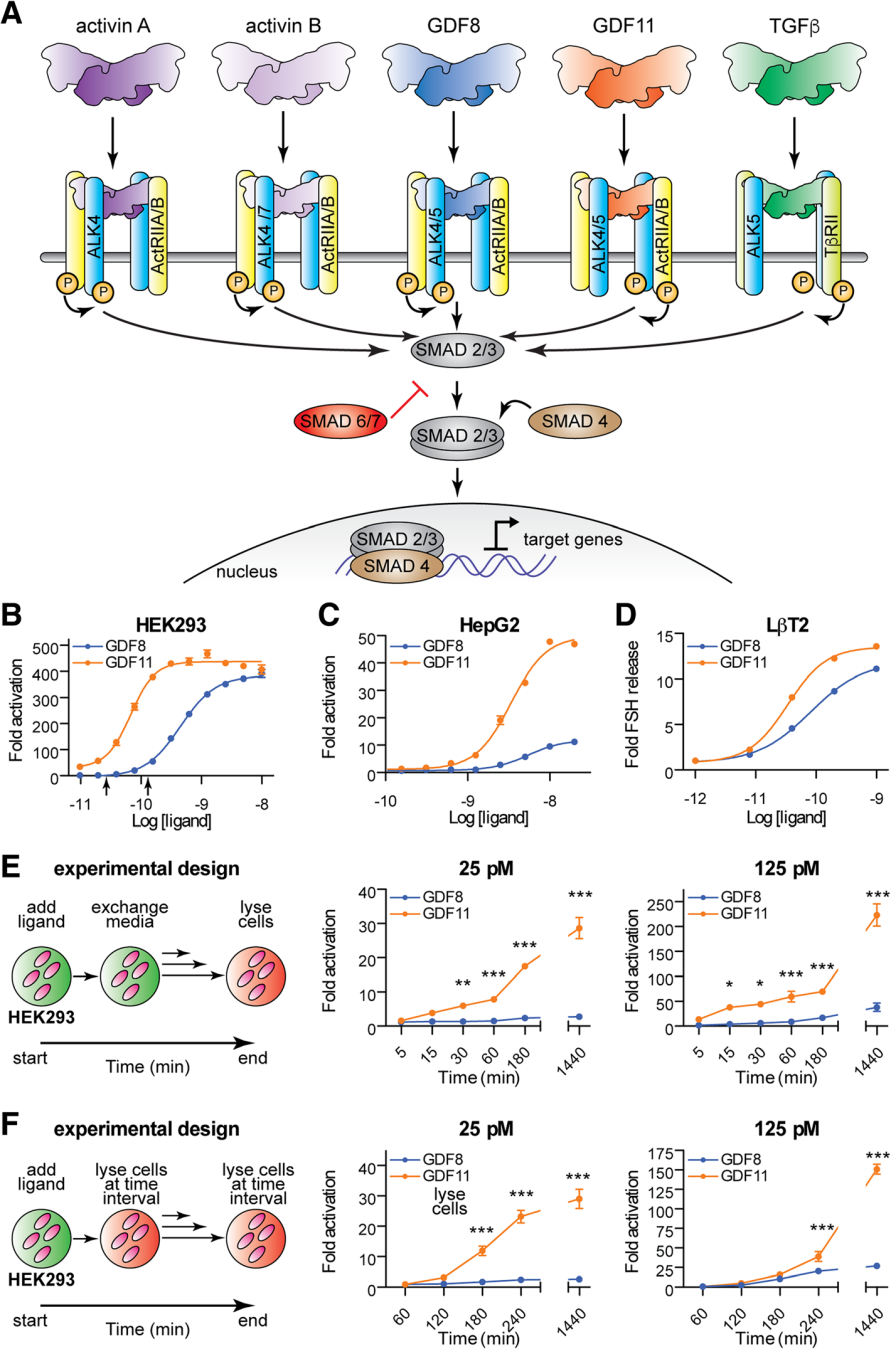
GDF11 is a more potent ligand than GDF8. **a** Overview of the well-established canonical activin A, activin B, GDF8, GDF11, and TGFβ receptor utilization and downstream SMAD pathway. **b**, **c**, **d** Potency differences between GDF8 and GDF11. Luciferase reporter gene assay ((CAGA)_12_ promoter) following titration of GDF8 (*blue*) and GDF11 (*orange*) ligands in HEK293 (**b**) and HepG2 (**c**) cells. Luciferase activity was assessed 18–24 h post ligand treatment. The *black arrows* in (**b**) indicate the ligand concentrations utilized in panels **e** and **f**. In **d**, mouse gonadotrope (LβT2) cells were treated with increasing doses of GDF8 (*blue*) or GDF11 (*orange*). Follicle-stimulating hormone (*FSH*) release was measured 24 h later, as previously described [92]. Refer to Additional file 1: Table S1 for a corresponding analysis of the activation curves. **e** Short exposure to GDF11 results in a significantly enhanced SMAD3-dependent response compared to GDF8. The experimental design (*left*) is such that the ligand was added to HEK293 cells stably transfected with the (CAGA)_12_ promoter driving the luciferase gene for the indicated time followed by replacement of media without ligand. Activity was measured 24 h after initial treatment. Cells were treated with GDF8 or GDF11 at a ligand concentration of 25 pM (*middle*) and 125 pM (*right*). **f** Time-dependent differences in the SMAD3 activation by GDF8 and GDF11. Similar experimental design (*left*) as in **e**, but instead cells were lysed and assessed for luciferase activity at the indicated time of ligand treatment. Cells were treated with GDF8 or GDF11 at a ligand concentration of 25 pM (middle) and 125 pM (right). Data information: In **b**, **c** and **e**, **f**, data are presented as fold activation above background (0 nM ligand concentration). Each concentration was performed in triplicate and shown as the mean ± standard error of the mean (*SEM*) of three independent experiments. In **d**, data are presented as fold FSH release above background (0 nM ligand concentrations). Each concentration was performed in triplicate and shown as the mean ± SEM of one experiment; data shown are representative of 10 independent experiments. In **e**, **f**, curves were compared using two-way ANOVA with Bonferroni correction (*P ≤ 0.05, **P ≤ 0.01, and ***P ≤ 0.001). Ligand sources: for **b**, **e**, **f**, gift from Acceleron Pharma; for **c**, **d**, purchased from R&D Systems; Cat. no. 788-G8-CF and Cat. no. 1958-GD-010-CF)

Similar to other TGFβ ligands, GDF8 and GDF11 are disulfide-linked dimers that are initially synthesized as precursors, which are cleaved by furin-like proteases to separate the N-terminal prodomain from the C-terminal mature domain [6, 18, 34]. Unlike most TGFβ ligands, mature GDF8 and GDF11 remain tightly bound to their prodomains, holding them in a latent state [9, 34–37]. Ligand activation requires additional cleavage of the prodomain by BMP1/tolloid (TLD) metalloproteinases [9, 34–37]. The ligand dimer elicits signal transduction by symmetrically binding two type II and two type I transmembrane serine/threonine kinase receptors (reviewed in [38]). Ligand-induced receptor clustering leads to phosphorylation of SMAD2 and SMAD3 (SMAD2/3) transcription factors by the type I receptor. Subsequent accumulation of SMAD2/3 in the nucleus results in activation or repression of GDF8 and GDF11 responsive genes (Fig. 1a) [6–8]. Similar to other ligands in the activin/inhibin subclass, GDF8 and GDF11 predominantly signal through the type II receptors, activin receptor kinase IIA (ActRIIA; ACVR2A) and ActRIIB (ACVR2B) and the type I receptors, activin-like receptor kinase 4 (ALK4; ACVR1B) and ALK5 (TβRI; Fig. 1a) [6–8]. There is also evidence that GDF11 can signal using the type I receptor ALK7 (ACVR1C) [8]. Furthermore, signaling by both GDF8 and GDF11 is controlled by extracellular protein antagonists, including FS [6, 39], FSTL3 [9], GASP1, and GASP2 [10, 40–42].

There have been conflicting reports comparing the biological functions of GDF8 and GDF11, with some question as to whether the two ligands are functionally equivalent [16, 17, 20–23, 43, 44]. Specifically, GDF11 was identified as a circulating factor that declines with age, and exogenous delivery of the mature ligand reversed many pathologies associated with aging [22, 23], including reducing age-related cardiac hypertrophy [22], improving skeletal muscle performance and repair [21], improving olfaction [20], and increasing neurogenesis [20]. Conversely, other reports have challenged these claims, arguing that GDF8 and GDF11 are functionally and biologically equivalent [16, 17, 43, 44]. However, it is difficult to compare the results, given that many of these studies did not utilize the same experimental design, sources of recombinant proteins, or strategies for detection of GDF8 and GDF11 proteins (recently reviewed in [5, 45]). Therefore, the extent of functional overlap between mature GDF8 and GDF11 without the prodomain remains to be determined. Furthermore, a rigorous side-by-side biochemical and biological comparison of mature GDF8 and GDF11 has yet to be performed to determine if distinct features exist between these growth factors.

In this study, using a variety of in vitro and in vivo systems, we show that GDF11 is significantly more potent than GDF8 and that the enhanced activity is due to differences in type I receptor utilization. In addition, we present three new X-ray crystal structures of apo-GDF8, apo-GDF11, and GDF11 in complex with follistatin 288 (FS288). These structures, including an additional recently solved apo-GDF11 crystal structure [46], reveal that differences between GDF11 and GDF8 cluster in the type I receptor binding epitope. Intriguingly, our structural analysis has revealed unique and alternate conformations of both GDF8 and GDF11, suggesting that both ligands are inherently flexible. GDF8/GDF11 chimeras, in which particular GDF11 residues are substituted into the GDF8 sequence, show that increased potency can be conferred to GDF8. Taken together, our results demonstrate that, despite the high sequence identity between mature GDF8 and GDF11, the ligands indeed possess different signaling properties.

## Results

### GDF11 is a more potent ligand than GDF8

Recent reports have attributed unique in vivo properties to GDF11 relative to GDF8 [20–23]. Therefore, we first aimed to determine if signaling differences exist between these two growth factors using HEK293 (Fig. 1b) and HepG2 (Fig. 1c) cells stably or transiently transfected, respectively, with the SMAD3-responsive (CAGA)_12_ luciferase reporter [47–51]. Side-by-side titration of mammalian-derived (see “Methods”) ligands revealed that GDF11 was more potent than GDF8 in both cell lines (Fig. 1b and c; Additional file 1: Table S1). Remarkably, we determined that the half-maximal effective concentration 50 (EC_50_) values for GDF11 in HEK293 and HepG2 cells were 0.08 nM and 3.4 nM, respectively, compared to 0.48 nM and 5.4 nM for GDF8, respectively (Fig. 1c; Additional file 1: Table S1). Interestingly, the maximal SMAD3 response achieved by GDF11 was ~ fourfold higher compared to GDF8 in HepG2 cells (Fig. 1c; Additional file 1: Table S1), suggesting that there may be differences in ligand potency and/or in the repertoire of receptors and their relative utilization. Furthermore, we found that GDF11 (EC_50_ 0.03 nM) more potently stimulated the release of follicle-stimulating hormone (FSH) than GDF8 (EC_50_ 0.08 nM) in murine LβT2 pituitary gonadotrope cells (Fig. 1d; Additional file 1: Table S1). Finally, GDF11 activated SMAD3 with ~ a sevenfold lower EC_50_ compared to GDF8 (6.5 nM versus 45.7 nM, respectively) in A204 cells transfected with the (CAGA)_12_ luciferase reporter (Ravindra Kumar, personal communication). These results are consistent with previous reports, which likewise indicated that GDF11 is a more potent activator of SMAD3-dependent signaling than GDF8 [16, 44]. Note that ligands from different sources were utilized and were directly compared using the SMAD3-responsive (CAGA)_12_ luciferase reporter assay (Additional file 2: Figure S1). For clarity, we have indicated the ligand sources utilized for each study in the figure legends.

Given that GDF11 stimulated greater SMAD3 activation in a concentration-dependent fashion compared to GDF8, we wanted to determine if this difference was maintained when we performed a pulse-chase exposure to each ligand (Fig. 1e and f; Additional file 3: Figure S2). To examine this, we performed two separate experiments. In the first, we treated HEK293 cells with GDF8 or GDF11 at three different concentrations (25, 125, and 620 pM) for the indicated times (5–180 min), after which the culture medium was removed and replaced with ligand-free culture medium for the duration of the experiment. At 24 h (1440 min), the cells were lysed and assayed for SMAD3-dependent luciferase activity (Fig. 1e; Additional file 3: Figure S2). GDF11 elicited a stronger response compared to GDF8 in both a concentration- and a time-dependent manner. In fact, at lower concentrations and exposure times, treatment with GDF11, but not GDF8, resulted in productive signaling (25 pM, Fig. 1e). A second experiment was performed where the cells were treated for the indicated time (60–1440 min) and immediately assayed for activity (Fig. 1f; Additional file 3: Figure S2). Interestingly, at the lower concentration (25 pM; Fig. 1f), we observed a significant difference in SMAD3 activation compared to GDF8 at most time points. These differences were less pronounced or non-existent at higher concentrations (Fig. 1f; Additional file 3: Figure S2). Together, these results indicate that the cellular response to mature GDF8 and GDF11 can be significantly different depending on the concentration and duration of exposure.

### Modulation of GDF8 and GDF11 activity by known extracellular antagonists

**Fig. 2:**
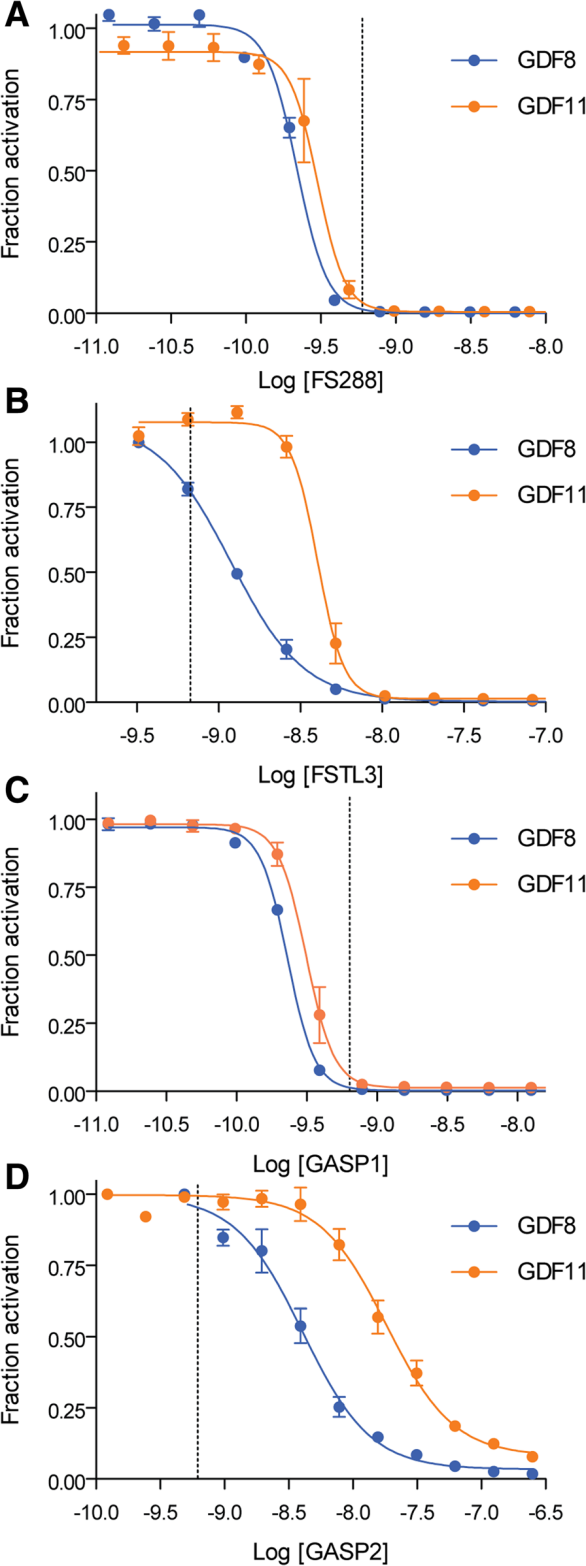
Inhibition of GDF8 and GDF11 by known antagonists. **a**, **b**, **c**, **d** Luciferase reporter assays showing the inhibitory activity following titration of FS288 (**a**), FSTL3 (**b**), GASP1 (**c**), and GASP2 (**d**) against a constant concentration (0.62 nM, *dashed bar*) of GDF8 (*blue*) or GDF11 (*orange*) in HEK293 (CAGA)_12_ cells. Refer to Table 1 for a corresponding analysis of the inhibition curves. Data information: In **a**–**d**, data are presented as fraction activation (ligand response at antagonist concentration/ligand response at 0 nM antagonist). Each concentration was performed in triplicate and shown as the mean ± SEM of two to three independent experiments. Data from independent experiments were combined and fit to non-linear regression with a variable slope. Ligand source: gift from Acceleron Pharma

Like many TGFβ ligands, extracellular antagonists modulate GDF8 and GDF11 signaling. Here, we determined if there were differences in the effectiveness of the known antagonists FS288, FSTL3, GASP1, and GASP2 to inhibit GDF8 or GDF11 using our HEK293 (CAGA)_12_ luciferase assay. FS288 and GASP1 similarly inhibited GDF8 or GDF11 (Fig. 2a and c and Table 1). However, FSTL3 and GASP2 (Fig. 2b and d and Table 1) more potently inhibited the actions of GDF8 relative to GDF11 (Table 1). Curiously, inhibition by FSTL3 for GDF11 revealed a more negative Hill slope compared to GDF8, suggesting that, while they are both potently inhibited, the binding interactions between the ligands and antagonists may not be identical.

**Table 1:** Analysis of known antagonists to GDF8 and GDF11 by luciferase reporter assay

Ligand	Antagonist	IC_50_ (nM)	IC_50_ (nM) 95% CI^*a*^	Log[IC_50_(M)] ± SEM^*b*^	Hill slope	Hill slope 95% CI
GDF8	FS288	0.2	0.21 to 0.23	−9.7 ± 0.01	−4.31	−5.17 to −3.43
GDF11		0.3	0.27 to 0.34	−9.5 ± 0.03	−4.77	−6.73 to −2.81
GDF8	FSTL3	1.2	1.08 to 1.30	−8.9 ± 0.02	−1.93	−2.19 to −1.68
GDF11		4.0	3.72 to 4.35	−8.4 ± 0.02	−5.41	−6.79 to −4.02
GDF8	GASP1	0.2	0.25 to 0.24	−9.6 ± 0.01	−4.47	−4.90 to −4.03
GDF11		0.3	0.29 to 0.39	−9.1 ± 0.02	−4.34	−5.49 to −3.20
GDF8	GASP2	4.1	3.31 to 5.07	−8.4 ± 0.05	−1.64	−2.15 to −1.14
GDF11		18.3	14.98 to 22.46	−7.7 ± 0.04	−1.67	−2.18 to −1.16

^*a*^ *CI* confidence interval

^*b*^ *SEM* standard error of the mean

### Structure of GDF11 bound to FS288

**Fig. 3:**
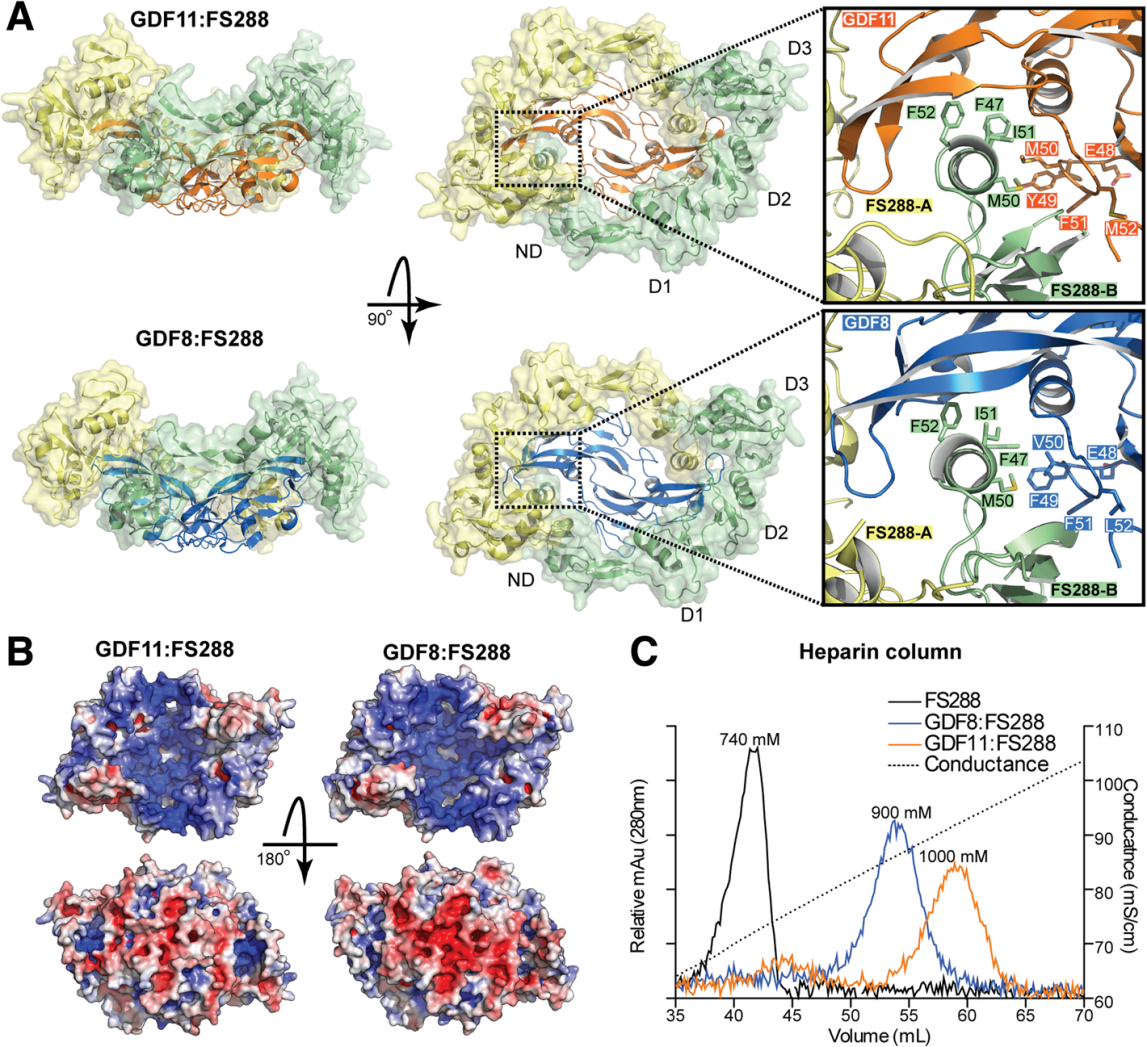
The structure of the GDF11:FS288 complex. **a** Comparison of the GDF11:FS288 (*top*) and GDF8:FS288 (*bottom*; Protein Data Bank (PDB) 3HH2; [54]) structures. The *inset* shows the differences between the interaction of the FSND helix and the ligand type I receptor epitope. Recombinant human GDF11 was obtained from Acceleron Pharma for resolution of this crystal structure. **b** Isoelectric surface representation of the GDF11:FS288 (*left*) and GDF8:FS288 (*right*) structures. Surfaces are colored *blue* (*positive*) and *red* (*negative*) on a scale of –5 to 5 k_b_T/e_c_ using the APBS plugin [97] for PyMol. **c** Heparin affinity analysis of FS288 alone, GDF11:FS288, and GDF8:FS288 complexes. GDF11:FS288 complex has higher affinity for heparin than FS288 alone indicated by elution at a higher ionic strength. Purified proteins and complexes (100 μg) were applied to a heparin column and eluted with a linear sodium chloride (NaCl) gradient. The approximate NaCl concentration for protein elution is shown at the peak maxima. Ligand source: gift from Acceleron Pharma

The complex of the GDF11 dimer bound to two molecules of FS288 was resolved using X-ray crystallography to 2.35 Å (Fig. 3a and Table 2). This is the first structure of GDF11 bound to a known antagonist. Similar to previous ligand:follistatin structures [52–54], two molecules of FS288 bind symmetrically to wrap around the GDF11 dimer occluding both type II and type I receptor binding sites. As expected, follistatin domains 1 (D1) and D2 overlap with the type II binding epitope, whereas the follistatin N-terminal domain (ND) occupies the type I binding slot. The overall structure of GDF11:FS288 is highly similar to that of the GDF8:FS288 complex (Fig. 3a; overall root-mean-square deviation (RMSD) = 0.657 Å). Nonetheless, the structure of GDF11:FS288 reveals minor changes in the positioning of residues in the helix of ND (Fig. 3a). For example, F47 of FS288 is pushed inward to accommodate the larger side chain of M50 in GDF11 versus V50 in GDF8 (Fig. 3a). This is consistent with other ligand:FS structures, showing that the ND helix is quite plastic in its “molding” to accommodate ligand differences at the type I interface [47, 48, 52–55]. As observed in previous ligand:FS structures [52–54], the ND from one molecule interacts with D3 from the other follistatin in a head-to-tail fashion. Similar to GDF8:FS288 [54], the electrostatic surface potential of GDF11:FS288 depicts a large, continuous electropositive surface on one side of the complex. This is formed by the combination of the heparin binding motif of the two FS molecules with positively charged residues of GDF11 (Fig. 3b) [54]. As such, the GDF11:FS288 complex has increased affinity for heparin as compared to FS288 alone, and slightly stronger affinity than GDF8:FS288 (Fig. 3c) [54, 56–59].

**Table 2:** X-ray data collection and refinement statistics (molecular replacement)

	GDF11:FS288 (native)^*a*^	apo-GDF8 (native)^*a*^	apo-GDF11 (native)^*a*^
Data collection			
Space group	P 2_1_ 2_1_ 2_1_	P 2_1_ 2_1_ 2_1_	P 3_2_ 2 1
Unit cell dimension			
*a, b, c* (Å)	56.0, 59.1, 288.5	29.6, 77.7, 119.5	65.2, 65.2, 101.9
*α, β, γ* (°)	90, 90, 90	90, 90, 90	90, 90, 120
Wavelength (Å)	1.03321	0.97856	1.10537
Resolution (Å)	2.35 (2.43-2.35)	2.25 (2.32-2.25)	1.9 (1.94-1.9)
*R*_merge_	0.076 (0.519)	0.079 (1.102)	0.069 (0.706)
*R*_pim_	0.046 (0.311)	0.032 (0.436)	0.032 (0.316)
Mn (*I*/*σI*)^*b*^	10.0 (2.6)	18.2 (2.0)	10.4 (2.0)
CC_1/2_^*c*^	0.74	0.72	0.996
Completeness (%)	100.0 (100.0)	100.0 (100.0)	100.0 (100.0)
Redundancy	3.7 (3.8)	7.1 (7.3)	5.8 (5.9)
Refinement			
Resolution (Å)	41.28-2.35	36.96-2.25	49.37-1.9
No. reflections	40,978 (4,031)	13,776 (1,912)	20,166 (2,642)
*R*_work_ (%), *R*_free_^*d*^ (%)	20.3/24.7	23.4/27.7	21.7/25.8
No. of atoms (molecules)			
Protein	6047 (784)	1443 (187)	1395 (181)
Water	170 (170)	30 (30)	43 (43)
Citrate	91 (7)		
Phosphate	15 (3)		
2-Methyl-2,4-pentanediol		56 (7)	
B-factors (average, Å^2^)	60.0	65.1	60.3
RMS deviations			
Bond lengths (Å)	0.005	0.003	0.007
Bond angles (°)	0.960	0.951	0.853
Ramachandran plot			
Favored (%)	97.15	94.44	97.63
Allowed (%)	2.59	5.56	2.37
Outliers (%)	0.26	0.0	0.0
Clashscore^*e*^	4.18	6.83	3.33

^*a*^Values in parentheses are for highest resolution shell

^*b*^Mn(*I*/*σI*) is defined as < merged < *I*h >/sd (< *I*h >) > ≈ signal/noise

^*c*^CC_1/2_ for highest resolution shell

^*d*^ *R*_free_ calculated from 5% of initial total number of reflections

^*e*^Determined by MolProbity

### GDF8 and GDF11 structural differences: follistatin-bound versus apo states

**Fig. 4:**
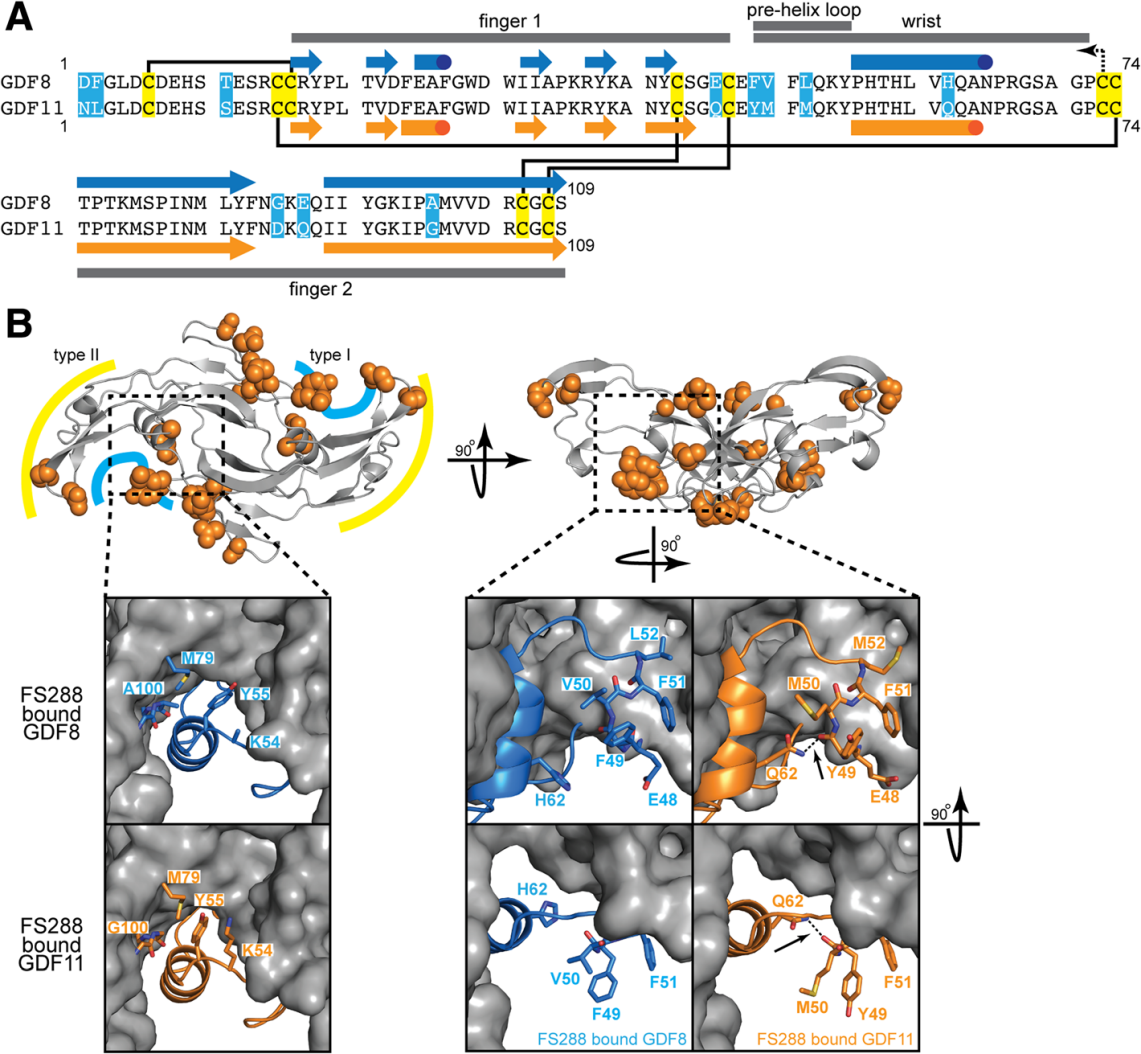
Sequence alignment and structural comparison of GDF8 and GDF11 in their FS288-bound state. **a** Sequence alignment of mature GDF8 and GDF11. Secondary structure for each ligand in the FS288-bound form is shown *above* and *below* the GDF8 and GDF11 sequences, respectively, where *arrows* represent β-sheet and *cylinders* represent α-helix. Cysteines are highlighted in *yellow*, and residues that are different between GDF8 and GDF11 are highlighted in *blue. Solid black lines* joining two cysteines indicate intramolecular disulfide bonds. The *dotted black line* indicates the cysteine responsible for the intermolecular disulfide bond. **b** Distribution of the amino acid differences between GDF8 and GDF11. GDF11 is shown as *ribbon*, and non-identical amino acids are shown as *orange spheres*. Symmetrical type I and type II interfaces are depicted with *blue* and *yellow lines* (*left*). *Insets* represent zoomed-in views depicting the molecular differences between FS288-bound GDF8 (*blue*) and FS288-bound GDF11 (*orange*) in the vicinity of the type I receptor binding interface. The *arrow* points to the hydrogen bond (*dotted line*) in the FS288-bound GDF11 between Q62 and carbonyl oxygen of Y49 (*middle*)

Initial investigation of the sequence alignment between GDF8 and GDF11 mature domains did not provide a clear explanation for why GDF11 is more potent than GDF8 (Fig. 4a). In other words, there did not appear to be major amino acid differences (i.e., charge reversals, inclusions/deletions, etc.) between the two ligands that would indicate significant differences in how they would interact with binding partners or receptors. However, when the differences between GDF8 and GDF11 were plotted on the structure, we saw that the majority are found near the type I receptor interface (Fig. 4b; Additional file 4: Figure S3). Therefore, to determine if there were any unique structural consequences of these amino acid differences, we made a more thorough examination of the X-ray crystal structures of mature GDF8 and GDF11 in the follistatin-bound and apo states.

**Fig. 5:**
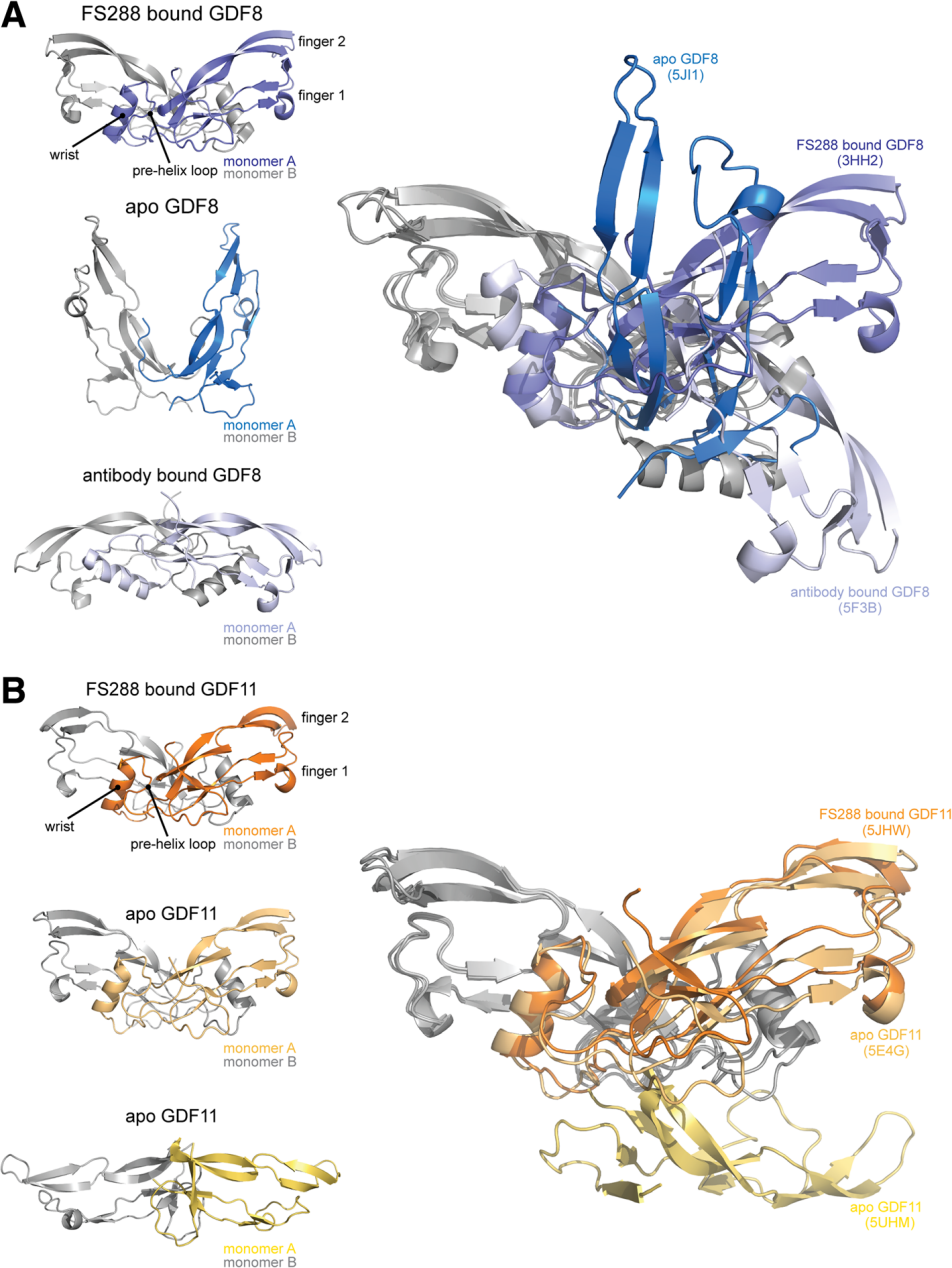
Structural comparison of GDF8 and GDF11 in FS288-bound and apo-conformations reveal ligand flexibility. Cartoon diagrams of the bound and apo states of GDF8 (**a**) and GDF11 (**b**). Each monomer is colored independently to highlight the differences in conformation. Superposition of each conformation using monomer_B_ (*gray*) is shown on the *left*. Protein Data Bank (PDB) IDs for bound ligands (FS288-bound GDF8 = 3HH2 [54]; antibody-bound GDF8 = 5F3B [61]; GDF11 = 5JHW) and apo ligands (GDF8 = 5JI1; GDF11 = 5E4G [46]; GDF11 = 5UHM). Resolution of apo-GDF8 was determined from recombinant human GDF8 produced and purified as previously described [54]. Resolution of FS288-bound and apo-GDF11 was determined from recombinant human GDF11 produced and purified by Acceleron Pharma

Superposition of the mature ligands from their respective FS288 complex structures did not reveal any major structural differences in the type II interface (convex surface) of the ligand. However, examination of the type I interface revealed two unique interactions in the wrist of GDF11 compared to GDF8 (Fig. 4a and b). The wrist region is known to be critically important for dictating ligand type I receptor affinity and specificity [38, 54, 60]. First, superposition of the ligands showed that Y55_A_ of GDF11 was shifted by ~1.3 Å inward toward the N-terminal side of the wrist helix on the adjacent monomer. This was facilitated by the lack of a side chain in G100_B_ of GDF11 (for clarity, the subscript is used to differentiate one ligand monomer from another; for reference*,* see Fig. 5a). In GDF8, the corresponding residue is A100_B_ where the methyl side chain sterically occludes Y55_A_. This difference allows Y55_A_ to more intimately interact with the opposing chain and facilitates additional hydrophobic interactions with M79_B_ and the aliphatic side chain of K54_A_. The second unique feature of GDF11 is the formation of an additional hydrogen bond between the backbone (Y49_A_) of the pre-helix loop and Q62_A_ located on the C-terminal side of the wrist helix (Fig. 4b). In GDF8, the corresponding residue is H62_A_ and does not interact with the pre-helix loop in the FS288-bound GDF8 crystal structure (Fig. 4b). In fact, H62_A_ does not interact with the pre-helix loop in additional crystal structures of GDF8 bound to either FSTL3 [47] or a neutralizing antibody (Fig. 5a) [61]. Thus, amino acid differences between GDF8 and GDF11 impose unique intramolecular contacts in GDF11 that are not present in GDF8. Given that these changes occur at the type I interface, it is intriguing to speculate that they could contribute to differences in ligand activity. However, we cannot exclude the possibility that these newly identified interactions are a result of binding to FS288. To explore this possibility, we examined the X-ray crystal structures of apo-GDF8 and apo-GDF11 [46].

We resolved the structure of apo-GDF8 to 2.25 Å (Fig. 5a and Table 2) and the apo-GDF11 structure to 1.90 Å (Fig. 5b, bottom; Table 2) using X-ray crystallography, thus allowing a direct comparison to the recently published apo-GDF11 structure (Fig. 5b, middle) [46]. Unexpectedly, resolution of the apo-GDF8 structure revealed a unique conformation such that the fingertips of each monomer are positioned proximally in a “closed” conformation compared to the classical distal positioning or “open” conformation observed for many ligands, including structures of FS-bound GDF8 (Fig. 5a, top) [38, 47, 54]. While the structure of apo-GDF8 adopted an unexpected “closed” conformation, a similar conformation has been observed for activin A when in complex with ActRIIB [62]. In both cases, the wrist region is disordered and not resolved in the crystal structure (Fig. 5a). Our new apo-GDF11 structure, solved under similar crystallization conditions as apo-GDF8, adopted an exaggerated “open” conformation such that the fingers extend past the horizontal plane (Fig. 5b). This conformation shares a similar exaggerated “open” conformation to the crystal structure of GDF8 bound to a neutralizing antibody (Fig. 5a, bottom) [61]. However, the wrist region in our apo-GDF11 structure is also disordered and not resolved in the crystal structure, similar to apo-GDF8 (Fig. 5b, bottom). The previously published apo-GDF11 crystal structure adopts the classic “open” conformation with an ordered wrist helix that includes the additional interchain hydrogen bond between Q62_A_ and the pre-helix loop observed in the FS288-bound GDF11 crystal structure. Taken together, crystallization of both GDF8 and GDF11 in their apo states reveals intrinsic structural flexibility in these ligands.

### GDF11 signals more effectively through type I receptors than GDF8

**Fig. 6:**
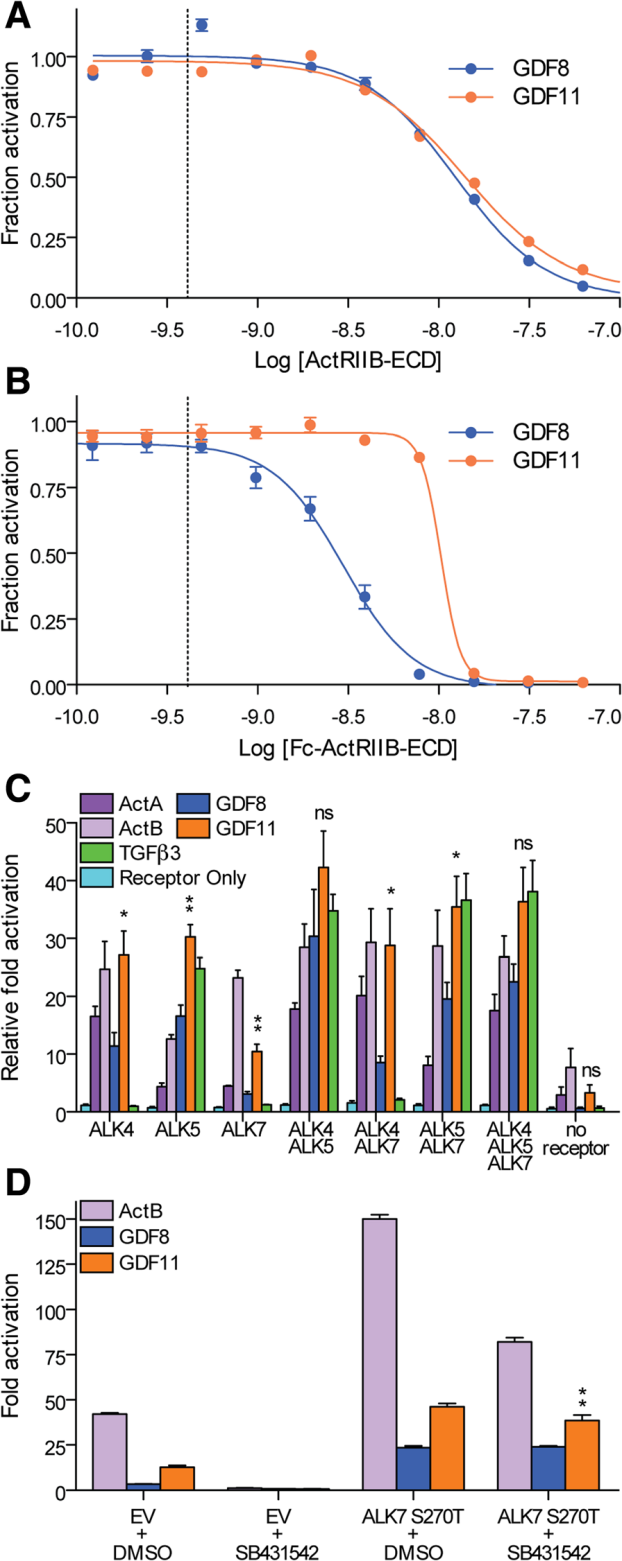
Enhanced type I receptor utilization by GDF11 compared to GDF8. **a**, **b**, Luciferase reporter assays showing the inhibitory activity following titration of ActRIIB-ECD (**a**), and Fc-ActRIIB-ECD (**b**) against a constant concentration (0.62 nM, *dashed bar*) of GDF8 (*blue*) or GDF11 (*orange*) in HEK293 (CAGA)_12_ cells. Refer to Table 3 for a corresponding analysis of the inhibition curves. **c** Differences in type I receptor utilization by GDF11 following transient transfection of a single type I receptor (ALK4, ALK5, or ALK7) or a combination of type I receptors (ALK4/ALK5, ALK4/ALK7, ALK5/ALK7, or ALK4/ALK5/ALK7). RIB L17 cells were co-transfected with 1.25 ng of the individual receptor DNA alone or various receptor DNA combinations (1.25 ng each receptor) and 2.5 ng (CAGA)_12_ promoter plasmid driving the luciferase gene. Purified recombinant ligands were added 12 h post transfection. Cells were then lysed and assessed for luciferase activity 8 h post ligand treatment. **d** Treatment of RIB L17 cells with DMSO or the type I receptor small molecule inhibitor SB431542 following transfection of empty vector (EV) or ALK7 S270T. RIB L17 cells were co-transfected with 1.25 ng of the individual receptor DNA and 2.5 ng (CAGA)_12_ promoter plasmid driving the luciferase gene. Purified recombinant ligands were added 12 h post transfection. Cells were then lysed and assessed for luciferase activity 8 h post ligand treatment. Data information: In **a**, **b**, data are presented as fraction activation (ligand response at antagonist concentration/ligand response at 0 nM antagonist). Each concentration was performed in triplicate and shown as the mean ± SEM of two independent experiments. Data from independent experiments were combined and fit to non-linear regression with a variable slope. In **c**, **d**, data are presented as fold activation defined as the total activation from each ligand compared to cells only transfected with the (CAGA)_12_ reporter construct. Each bar is the mean ± SEM. A representative experiment is shown of at least two independent experiments in which each concentration was performed in duplicate or triplicate. Only comparisons between GDF8 and GDF11 were made. *P ≤ 0.05 and **P ≤ 0.001 (Student’s *t* test). Ligand sources: GDF8 and GDF11, gift from Acceleron Pharma; Activin A, Activin B, and TGFβ3, produced and purified as described in “Methods”

To better understand the potency differences between GDF11 and GDF8, we compared their receptor binding affinities and utilization. Given that the structural differences between GDF11 and GDF8 are positioned at the type I receptor interface, we hypothesized that potency differences are likely through differences in type I interactions. In order to rule out potential differences in type II receptor binding, we first compared the inhibitory potential (i.e., dominant negative effect) of the soluble receptors, ActRIIB extracellular domain (ECD) and Fc-ActRIIB-ECD using our HEK293 (CAGA)_12_ luciferase assay (Fig. 6 and Table 3). We found no difference in the IC_50_ value for ActRIIB-ECD between GDF8 and GDF11 (12.4 nM versus 14.0 nM, respectively), whereas Fc-ActRIIB-ECD was ~ threefold more potent in inhibiting GDF8 (IC_50_ = 3.0 nM) than GDF11 (IC_50_ = 10.3 nM; Fig. 6a and b; Table 3). While the calculated Hill slope for the ActRIIB-ECD was similar for GDF8 and GDF11, we observed a more negative Hill slope for the inhibition of GDF11 by the Fc-ActRIIB-ECD compared to GDF8, similar to our earlier results showing a more negative Hill slope when GDF11 was antagonized by FS288, FSTL3, and GASP1 (Fig. 2 and Table 1).

**Table 3:** Analysis of ActRIIB-ECD on GDF8 and GDF11 activity by luciferase reporter assay

Ligand	Receptor	IC_50_ (nM)	IC_50_ (nM) 95% CI^*a*^	Log[IC_50_(M)] ± SEM^*b*^	Hill slope	Hill slope 95% CI
GDF8	ActRIIB-ECD^*c*^	12.4	12.24 to 13.66	−7.9 ± 0.02	−1.73	−1.99 to −1.47
GDF11		13.0	12.61 to 15.46	−7.9 ± 0.02	−1.57	−1.79 to −1.35
GDF8	Fc-ActRIIB-ECD	3.0	2.60 to 3.38	−8.5 ± 0.03	−2.24	−2.82 to −1.65
GDF11		10.3	9.40 to 11.125	−8.0 ± 0.02	−8.05	−10.46 to −5.63

^*a*^ *CI* confidence interval

^*b*^ *SEM* standard error of the mean

^*c*^ *ECD* extracellular domain

Next, we determined if GDF8 and GDF11 signal through similar type I receptors. Numerous studies have shown that GDF8 and GDF11 utilize the type I receptors ALK4 and ALK5 [6–8]. In addition, GDF11 can signal through ALK7 [8], although it is unclear if GDF8 also utilizes this receptor. To our knowledge, a study directly comparing signaling by both ligands via the three receptors has not been reported. Therefore, we assessed the relative (CAGA)_12_ luciferase reporter response to GDF8 or GDF11 following transient expression of individual type I receptors in RIB L17 cells (Fig. 6c and d). This cell line lacks ALK5 expression and expresses low levels of ALK4 [7, 54, 63]. The level of ALK7 expression in these cells has not been reported. As expected, activin A robustly signaled in cells transfected with ALK4 [63, 64], activin B signaled through both ALK4 and ALK7 [65, 66], and TGFβ3 specifically signaled through ALK5 (Fig. 6c) [67, 68]. Consistent with previous reports, GDF8 and GDF11 readily signaled through ALK4 and ALK5 [6–8, 54]. Additionally, we confirmed that GDF11 signaled through ALK7 (Fig. 6c and d) [8]. Strikingly, GDF11 induced greater reporter responses than GDF8 via ALK4, ALK5, or ALK7 (Fig. 6c and d). We should note that activin A, activin B, GDF11, and to a lesser extent GDF8, stimulated some reporter activity in the absence of exogenous receptors, suggesting low levels of endogenous ALK4 and ALK7 expression by these cells (Fig. 6c). Consistent with this idea, activin B, GDF8, and GDF11 induction of reporter activity was completely blocked by addition of the ALK4/5/7 small molecule inhibitor SB431542 (Fig. 6d) [69–72]. Importantly, signaling could be rescued by transfection of an SB431542-resistant form (S270T) of human ALK7 (Fig. 6d), confirming that all three ligands can signal via ALK7 with relative potencies of activin B > GDF11 > GDF8. Collectively, our results indicate that GDF11 can generate a greater SMAD3-dependent signal compared to GDF8 through all three type I receptors.

Given that type I receptor expression is not ubiquitous in all cells, we next wanted to determine if differences in ALK4/5/7 combinations would further distinguish or normalize response differences between GDF8 and GDF11. Different receptor combinations modestly enhanced signaling relative to individual receptors (Fig. 6c). Interestingly, when ALK4 and ALK5 were co-transfected, there was no statistically significant difference in response to GDF8 and GDF11 (at the concentration tested), in contrast to when either receptor was expressed alone (Fig. 6c). Co-transfection of ALK4 and ALK7 resulted in a more robust response to GDF11 over GDF8. Taken together, these results further suggest that ligand sensitivity can be driven, in part, by the receptor(s) expressed on a particular cell type, thereby providing a possible avenue by which cells may discriminate between highly similar ligands, such as GDF8 and GDF11.

**Fig. 7:**
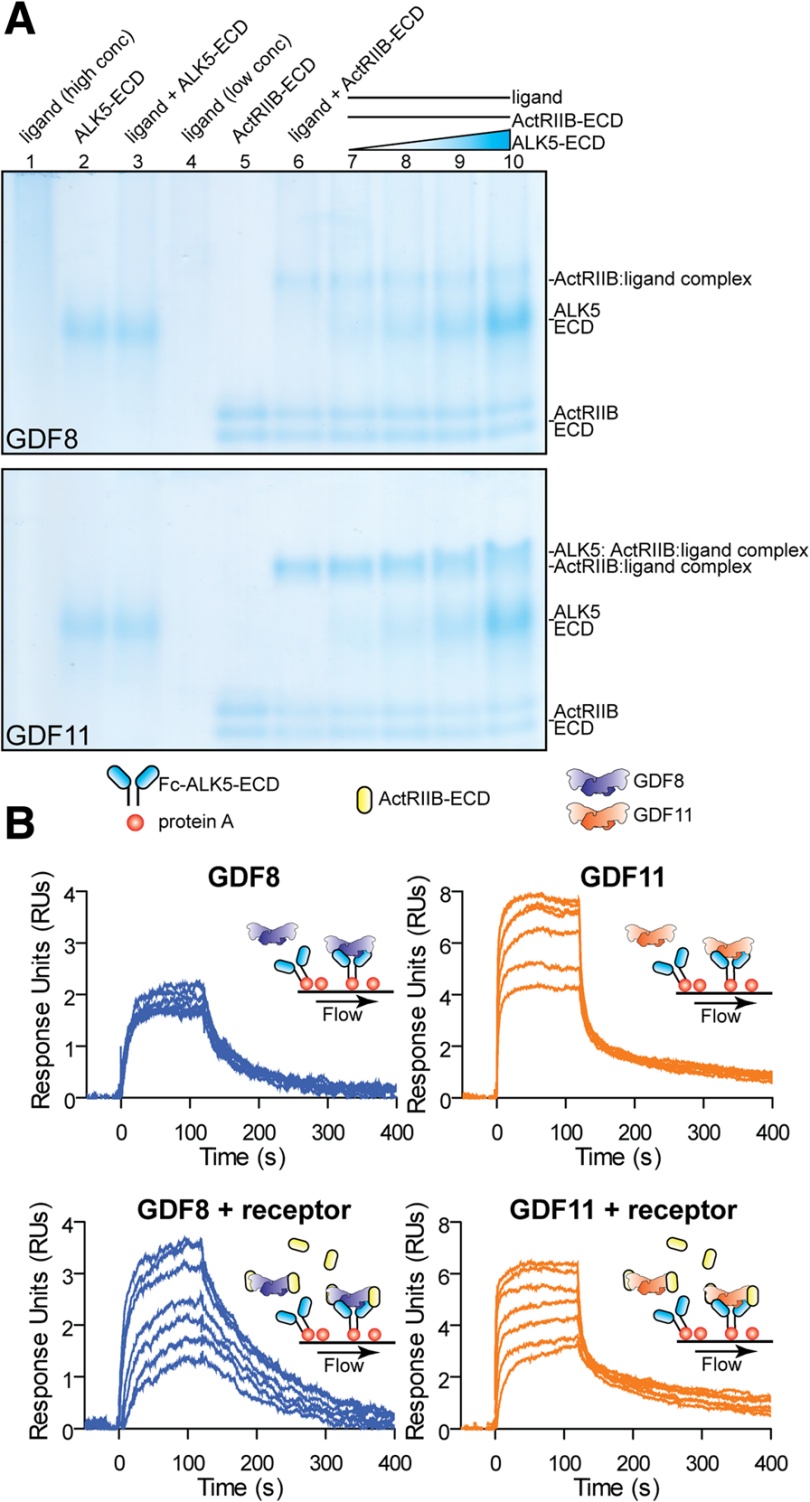
Binding of GDF11 to the type I receptor ALK5. **a** Basic Native-PAGE (12%) of GDF8 (*top*) and GDF11 (*bottom*) ligand, receptor, and ligand:receptor complexes. To test for ligand-receptor complex formation, receptors were first diluted into 20 mM HEPES pH 7.4 followed by addition of the ligand. The ligand:receptor mixtures were held at room temperature (25 °C) for 15 min before being loaded and run in the gel. Amount loaded for each protein: ligand (high conc.; lane 1), ALK5-ECD (lane 2), ligand + ALK5-ECD (lane 3), ActRIIB-ECD (lane 5) = 3 μg each; ligand (low conc.; lane 4) = 1.5 μg; ligand + ActRIIB-ECD (lane 6) = 1.5 μg ligand and 3 μg ActRIIB-ECD; for ALK5-ECD titration (*blue triangle*; lanes 7–10) ligand = 1.5 μg, ActRIIB-ECD = 3 μg, and 1, 2, 4, or 8 μg ALK5-ECD. Proteins were visualized by colloidal Coomassie stain. **b** Surface plasmon resonance (SPR) sensorgrams of GDF8 (*left*) and GDF11 (*right*) binding to Fc-ALK5-ECD. Fc-ALK5-ECD was captured by Protein A that was amine coupled to a CM5 chip. Serial dilutions (500–15.265 nM) of each ligand alone (*top*) or in the presence (*bottom*) of a twofold molar excess of ActRIIB-ECD were allowed to associate for 120 s at a 70 μL/min flow rate followed by a 280 s dissociation at 37 °C. Sensorgrams were double referenced using an average of two 0 nM ligand injections. Data information: Ligand sources: GDF8 and GDF11, gift from Acceleron Pharma; Activin A, Activin B, and TGFβ3, produced and purified as described in “Methods”

Structural studies describing the interaction between the type I receptors and the activin/inhibin subclass are lacking, predominantly due to the inability to generate recombinant receptors whose folding and biological activity can be confirmed unequivocally. While this barrier remains unresolved for ALK4 and ALK7, we can reliably study the interactions between ligands and the ALK5-ECD [73, 74]. We tested binding of GDF8 and GDF11 to recombinant ALK5-ECD in the absence or presence of ActRIIB-ECD using Native-PAGE and surface plasmon resonance (SPR; Fig. 7). Neither mature GDF8 nor GDF11 alone were readily resolved using Native-PAGE (lanes 1 and 4 in Fig. 7a). However, addition of ActRIIB-ECD to either ligand resulted in a mobility shift compared to ActRIIB-ECD alone, indicative of complex formation between the ligand and type II receptor (compare lanes 5 and 6 in Fig. 7a). Mixing the ligands with ALK5-ECD alone did not result in a detectable ALK5-ECD-ligand complex (lanes 2 and 3 in Fig. 7a). However, when we co-incubated either ligand with a constant amount of ActRIIB-ECD followed by titration with ALK5-ECD, we observed a noticeable shift in the migration pattern for GDF11, especially apparent at excess molar ratios of ALK5-ECD. Under similar conditions, little to no change was observed with GDF8 (compare lanes 6 through 10 in Fig. 7a). Although high concentrations were required, these results suggest that a low-affinity ternary complex composed of GDF11:ActRIIB:ALK5 was more readily formed than the corresponding complex with GDF8.

To clarify these results, we attempted to detect direct binding of either ligand to ALK5-ECD, Fc-ALK5-ECD, and Fc-ActRIIB using SPR (Fig. 7b; Additional file 5: Figure S4). In this experiment, the receptor was immobilized on the SPR biosensor chip with standard amine coupling or captured using Protein A. As expected, both ligands readily bound the Fc-ActRIIB-ECD (Additional file 5: Figure S4D) [6–8]. Qualitatively, GDF11 interacted with both ALK5-ECD and Fc-ALK5-ECD in a dose-dependent manner and exhibited a fast association and rapid dissociation (Fig. 7b; Additional file 5: Figure S4A–C, E, F). In contrast, GDF8 did not bind to ALK5-ECD or Fc-ALK5-ECD in a dose-dependent fashion (Fig. 7b; Additional file 5: Figure S4E, F). Given that ALK5 binding to TGFβ is significantly enhanced through a cooperative interaction with TβRII [73, 74], we speculated that ActRIIB might improve GDF8 or GDF11 binding to ALK5-ECD. Interestingly, co-injection of ActRIIB-ECD with GDF11 did not appear to alter the association or dissociation with ALK5-ECD compared to GDF11 alone (Fig. 7b right two panels; Additional file 5: Figure S4B, C). However, we did observe a detectable improvement in binding of GDF8 to ALK5-ECD in the presence of ActRIIB-ECD (Fig. 7b; Additional file 5: Figure S4B, C). These results suggest that GDF11 binding to ALK5 is likely more favorable compared to GDF8. However, it is clear that additional experiments will be necessary to fully resolve the interactions between these ligands and their cognate type I receptors.

### Substitution of GDF11-like residues into GDF8 enhances ligand activity

**Fig. 8:**
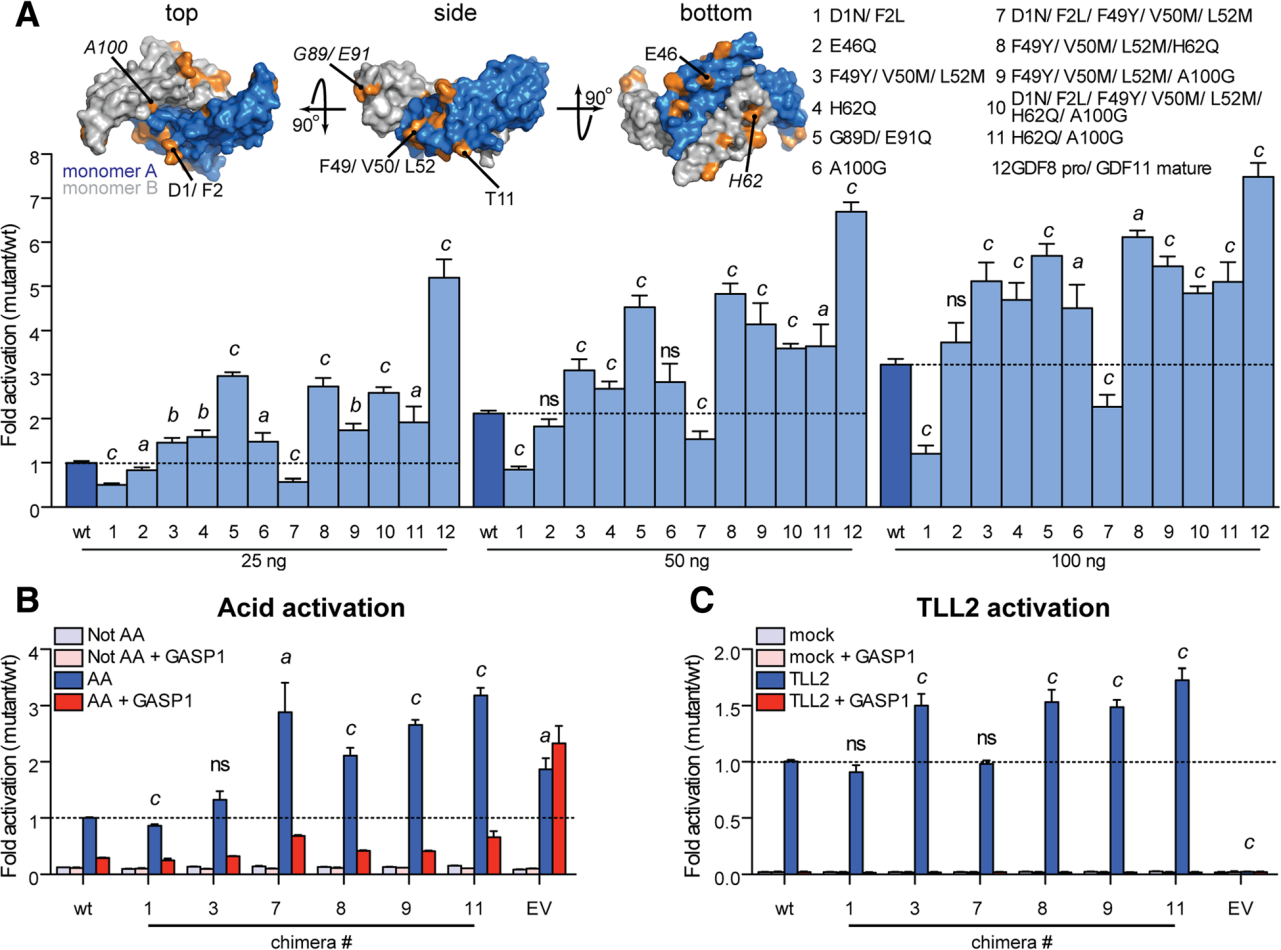
Generation of GDF8/GDF11 chimeric ligand confers potency to GDF8. **a** Luciferase reporter gene assay ((CAGA)_12_ promoter) following transient co-transfection of HEK293 cells with GDF8/GDF11 chimera constructs (25 ng, 50 ng, or 100 ng), furin (50 ng), and TLL2 (50 ng). All constructs contain human wild-type GDF8 prodomain followed by human wild-type or mutated mature GDF8. 18–24 h post transfection, the culture media were replaced with serum-free media and allowed to incubate for an additional 24 h, at which point the cells were lysed and measured for luciferase activity. wt = GDF8; point mutations are indicated by number. For chimera number 12 (GDF8 pro/GDF11 mature), each non-identical residue between GDF8 and GDF11 was mutated to generate the equivalent wt GDF11 ligand. A surface representation of GDF8 dimer is shown. Each monomer is colored independently (monomer A = *blue*; monomer B = *gray*). Non-identical residues are shown in *orange*. **b**, **c** Luciferase reporter gene assay ((CAGA)_12_ promoter) following exogenous addition of purified empty vector, wt, or GDF8/GDF11 chimeric latent protein complexes (see Additional file 6: Figure S5). The latent complexes (~1.5 ng mature ligand) were activated by treatment with acid (**b**; adjusted to pH 2.5 for 1 h and then neutralized), or the HEK293 cells were transiently transfected with TLL2 prior to protein addition (**c**). *AA* acid activated, *Not AA* no acid activation, *Not AA + GASP1* no acid activation but complexes added to cells in the presence of 100 nM GASP1, *AA + GASP1* acid activation in the presence of 100 nM GASP1, *EV* empty vector transfected, *EV + GASP1* empty vector transfected, but complexes added in the presence of 100 nM GASP1, *TLL2* transfected with TLL2, *TLL2 + GASP1* cells transfected with TLL2, complexes added in the presence of 100 nM GASP1. Data information: In **a**, **b**, **c**, data are presented as a ratio of the fold activation (mutant/wt GDF8) where each was normalized to the response of empty vector control. Each concentration was performed in duplicate or triplicate, and each bar is shown as the mean ± SEM from two to three independent experiments. ^*a*^ *P* ≤ 0.05, ^*b*^ *P* ≤ 0.01, ^*c*^ *P* ≤ 0.001, and *ns* = not significant (Student’s *t* test). Ligand sources: produced and purified as described in “Methods”

To determine which residues are important for conferring enhanced activity to GDF11, we generated a number of GDF8/GDF11 chimera constructs. Site-directed mutagenesis was performed on the full-length human GDF8 to introduce GDF11-like substitutions (for reference, see Fig. 4a). We compared the activity of the chimeras to wild-type (wt) GDF8 by transient co-transfection of the constructs with tolloid-like 2 (TLL2) and furin using HEK293 (CAGA)_12_ luciferase cells (Fig. 8; Additional file 6: Figure S5). We focused our efforts on the regions showing the most divergence between GDF8 and GDF11, specifically the type I interface consisting of the pre-helix loop and wrist (for reference, see Additional file 4: Figure S3). Interestingly, transient expression of a number of chimeric mutants revealed a significant gain in activity (Fig. 8a). The increased activity was observed at nearly every concentration (25, 50, and 100 ng DNA) tested (Fig. 8a), with differences between the chimeric mutants and wt GDF8 more pronounced at lower concentrations (25 ng versus 100 ng; Fig. 8a). Thus, incorporation of GDF11-like residues into GDF8 can increase ligand potency. Consistent with our overall hypothesis, we found that full substitution (chimera 12, GDF8pro/GDF11 mature) of all different residues between mature GDF8 and GDF11 resulted in a very potent ligand compared to wt GDF8, particularly at lower concentrations (Fig. 8a). Remarkably, a significant gain in activity was observed in the chimeras that incorporated the GDF11 residues, Q62 and G100 (e.g., chimera 11 in Fig. 8a), which were shown in the structure to form additional, stabilizing interactions at the ligand dimer interface (Fig. 8a).

To rule out that activity differences were attributed to differences in the relative amounts of protein expression, we produced and purified wt (wt GDF8 prodomain:mature GDF8) and selected mutant latent complexes (wt GDF8 prodomain:mature GDF8/GDF11 chimera). To determine their specific activity relative to wt GDF8 (Fig. 8b and c), we used two methods known to activate the latent complex: acid activation (treatment with HCl to pH 2.5; [24]) and activation with a member of the TLD family, such as tolloid-like 2 (TLL2) [75]. Overall, our results from treatment with purified protein were consistent with the results from transient transfection (Fig. 8b and c). As expected, little to no luciferase activity was detected in cells treated with the latent complexes unless they were acid activated (Fig. 8b) or applied to cells transfected with TLL2 (Fig. 8c). In addition, we performed the experiments in the presence of GASP1, a specific antagonist of GDF8 and GDF11 [10, 42, 76], to confirm that the signal was specific to wt GDF8 or GDF8/GDF11 chimeras (Fig. 8b and c). Both activation methods revealed similar findings with the exception of the acid-activated empty vector sample (EV; conditioned medium from empty vector transfected and mock purified). The acid-activated EV sample showed a significant response that could not be attenuated with GASP1 (Fig. 8b), indicating possible contamination by additional ligands or pH-dependent artifacts. Despite this possibility, most of the signal with GDF8 or GDF8/GDF11 chimeras was attenuated by GASP1, suggesting that the majority of the signal was specific to the ligands added. Moreover, the higher background signal was not observed in the TLL2 experiment (Fig. 8c). Together, these data support the conclusions that mature GDF11 is more active than GDF8 and that GDF8 activity can be enhanced through substitution of specific residues from GDF11.

### GDF11 is a more potent inducer than GDF8 of phosphorylated SMAD2/3 in mouse primary skeletal myoblasts in vitro and the mouse myocardium in vivo

**Fig. 9:**
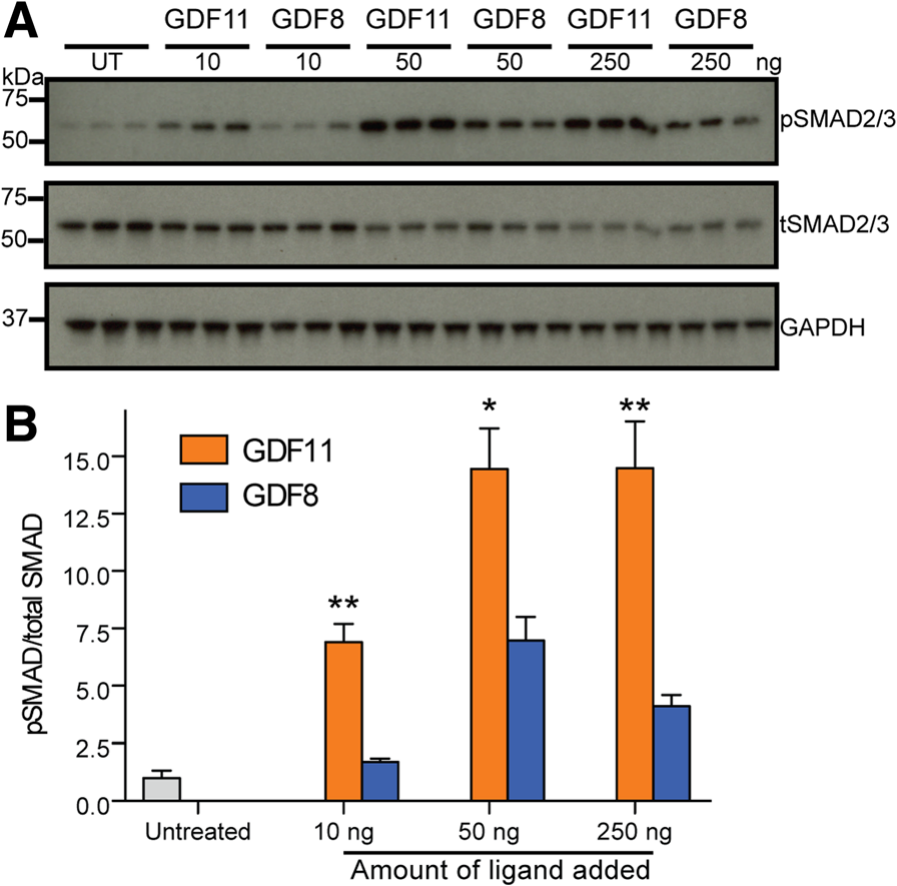
Exogenous treatment of GDF11 potently activates SMAD2/3 in cultured primary skeletal muscle myoblasts. **a**, **b** Western blot (**a**) and quantification (**b**) showing phosphorylated SMAD2/3 (*pSMAD2/3*), total SMAD2/3 (*tSMAD2/3*), and *GAPDH* of cultured primary skeletal muscle myoblasts following treatment with GDF8 or GDF11. Cells were treated with a range of ligand concentrations (10, 50, and 250 ng) for 1 h, lysed, and probed for the indicated proteins. 20 μg total protein loaded. Primary skeletal myoblasts were obtained from three different animals (*n* = 3). Data information: In **b**, data are presented as a ratio of pSMAD2/3 to total SMAD2/3. To obtain this ratio, pSMAD2/3 or SMAD2/3 was first normalized to GAPDH. The ratio of this quotient was then graphed. Data are presented as the mean ± SEM. *P ≤ 0.05 and **P ≤ 0.01 (Student’s *t* test). Ligand sources: PeproTech Cat. no. 120-00 and Cat. no. 120-11

Since we have shown that GDF11 is more potent than GDF8 in multiple cell lines, we next sought to determine if similar differences in potency could be seen in primary cells. Consistent with a previous report [16], western analysis of cultured myoblasts treated with mature GDF8 or GDF11 at a range of concentrations showed robust, dose-dependent induction of phosphorylated SMAD2/3 (pSMAD2/3) compared to untreated cultures (Fig. 9a). Further, GDF11 treatment elicited a significantly greater induction of pSMAD2/3 compared to equivalent concentrations of GDF8 (Fig. 9a and b).

**Fig. 10:**
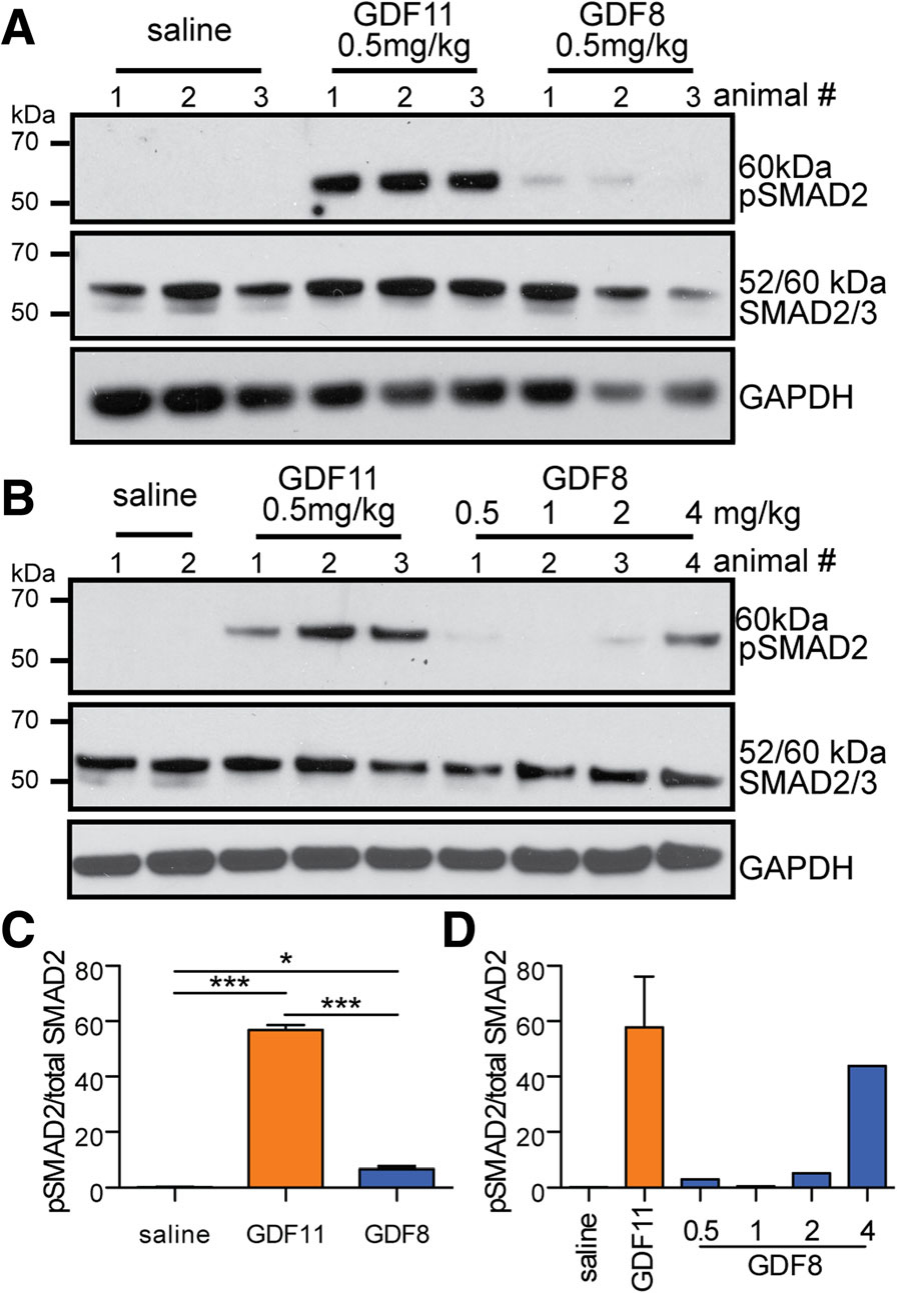
Intravenous injection of GDF11, but not GDF8, elicits robust phosphorylation of SMAD2/3 in the mouse myocardium. **a**, **b**, **c**, **d** Representative western blots (**a**, **b**) showing phosphorylated SMAD2 (*pSMAD2*), total SMAD2/3 (*SMAD2/3*), and GAPDH and corresponding quantification (**c**, **d**) in the mouse myocardium 1 h post tail-vein injection of saline, GDF8, or GDF11. **a**, **c** show a direct comparison in the level of pSMAD2 in the myocardium following injection of 0.5 mg/kg GDF8 or GDF11 (*n* = 3 mice/treatment). **b**, **d** show that nearly eightfold more GDF8 than GDF11 is required to achieve a similar pSMAD2 response (saline *n* = 2 mice; GDF11 = 0.5 mg/kg *n* = 3 mice; GDF8 *n* = 1 mouse per dose). Data information: In **c**, **d**, data are presented as a ratio of pSMAD2/total SMAD2/3. To obtain this ratio, pSMAD2 or SMAD2/3 was first normalized to GAPDH. The ratio of this quotient was then graphed. Data are presented as the mean ± SEM. *P ≤ 0.05 and ***P ≤ 0.001 (Student’s *t* test). Ligand sources: PeproTech Cat. no. 120-00 and Cat. no. 120-11

To determine if similar potency differences between GDF11 and GDF8 may also occur in vivo, we next examined SMAD2 phosphorylation (pSMAD2) in the mouse myocardium following intravenous (tail vein) injection of various doses of GDF8 and GDF11 (Fig. 10). pSMAD2 levels were significantly increased in the myocardium 1 h after injection of 0.5 mg/kg of GDF8 or GDF11 (Fig. 10a and c). At equivalent doses, GDF11 stimulated significantly more pSMAD2 than GDF8 (Fig. 10a and c). In fact, nearly eightfold more GDF8 than GDF11 was required to achieve similar levels of pSMAD2 (Fig. 10b and d). These data suggest that, depending on dose, intravenous GDF8 or GDF11 can result in a significantly different SMAD2/3-dependent response in the myocardium.

## Discussion

Previous studies indicate that GDF8 and GDF11 have both divergent and overlapping roles in developmental processes and tissue maintenance in adult animals (reviewed in [1–5]). While the role of GDF11 during development is well defined, its role in the adult remains poorly understood. However, recent evidence suggests that GDF11 is an important circulating factor that declines with age and exogenous treatment with GDF11 can reverse age-related effects including reducing cardiac hypertrophy [22, 23], improving skeletal muscle regeneration [21], and promoting neurogenesis [20]. However, other studies have contradicted certain aspects of these claims, fueling significant controversy on the functional similarities and differences between GDF11 and GDF8 [16, 17, 43, 44]. Controversy notwithstanding, in this study, we investigated the signaling activity and structural features of the mature GDF11 and GDF8 ligands.

At the outset of our studies, we made the unexpected observation that GDF11 is a more potent signaling ligand than GDF8. This observation was consistently and reproducibly observed across multiple laboratories, using different ligand preparations, in multiple cell lines and cultured primary cells, and recapitulated in vivo in the mouse myocardium. The current dogma in the literature suggests that there are no differences in the signaling potencies of GDF8 and GDF11 and that the ligands have identical signaling properties [16, 17, 43, 44]. Our results are consistent with this idea, but only at the highest ligand concentrations examined. Interestingly, prior studies also appeared to show that GDF11 is more potent or active than GDF8 [16, 44], yet many conclusions from these studies maintain the notion that GDF8 and GDF11 signal and function identically. The conclusion that GDF8 and GDF11 are indistinguishable may derive from the fact that subsequent comparisons were performed at concentrations that were above the EC_100_ for both ligands, thus resulting in identical transcriptional outcomes. In fact, we show that at high concentrations of GDF8 and GDF11, SMAD3-dependent readout is indistinguishable under certain conditions (see early time points in Fig. 1e and f). However, our data also reveal that GDF11 can elicit a significant response under conditions where GDF8 appears to have little to no activity. These results show that the responses generated by GDF8 and GDF11 are highly dependent on the concentrations of ligand and the available receptors, emphasizing that GDF8 and GDF11 are not equivalent. To be clear, these data neither support nor refute claims made by other groups regarding the functional outcomes resulting from GDF8 or GDF11 signaling. Rather, they demonstrate that the biochemical responses elicited by GDF8 and GDF11 at equivalent concentrations are significantly different under the conditions we tested. The physiological relevance, if any, which may result from potency differences, requires additional investigation.

Given the nearly identical sequence of their mature ligands, we were initially puzzled by the potency differences between GDF8 and GDF11, especially since most of the changes are relatively conserved. Therefore, we turned to X-ray crystallography to determine the structures of GDF11 in complex with FS288 in addition to GDF8 and GDF11 in their apo forms. While this manuscript was in preparation, an additional apo-GDF11 structure was resolved [46], thus providing the opportunity to compare multiple structures of each ligand in their FS288-bound and apo states. As expected, the overall structures of the ligands when bound to FS288 are highly similar with minor changes in the FSND helix of FS288, a region previously shown to be able to accommodate different ligands [47, 48, 52–55]. Comparison between GDF8 and GDF11 revealed that differences cluster within and around the type I receptor binding surface. In particular, differences are observed in residues within or bordering the wrist helix, an area important for type I receptor binding ([54]; reviewed in [38]).

To explore if these differences are the basis for increased activity, we used a chimeric approach that introduced GDF11-specific residues into GDF8. Apart from the pre-helix loop, the two most prominent differences between the structures of GDF11 and GDF8 are located at each end of the wrist helix and include GDF11 residues Q62 and G100. In both cases, substitution of the individual GDF8 residues H62 and A100 with the GDF11 residues Q62 and G100 resulted in increased activity over wild-type GDF8. Moreover, combination of these two mutations (H62Q/A100G) resulted in an additional gain in activity over the single mutations alone, suggesting an additive effect. In GDF11, these residues appear to stabilize the dimer interface where an additional hydrogen bond is formed between Q62 and the backbone of the pre-helix loop. Thus, increased dimer stability or stability of the interaction between the wrist and pre-helix loop might be one explanation for the increased ligand activity of GDF11, though we cannot rule out the possibility that these residues directly interact with the receptors. However, given that activity differences are observed over multiple receptors, it is more likely that potency differences are a result of inherent ligand differences (e.g., differences in ligand flexibility/conformation). Furthermore, introducing the GDF11-specific residues in the pre-helix loop (F49Y/V50M/L52M), a region known to be important for receptor specificity ([54]; reviewed in [38]), also results in increased activity. Additionally, introduction of GDF11 fingertip residues, D89 and Q91, into GDF8 also significantly increased ligand activity. However, our structural data do not provide a molecular explanation for how these variances confer differences in activity. Nevertheless, they do suggest that the potency differences between GDF8 and GDF11 are a result of a combination of the residue differences within and around the type I binding interface.

Resolution of the apo-GDF8 and apo-GDF11 crystal structures revealed that both ligands exhibit significant conformational flexibility. Interestingly and despite the high sequence identity, nearly identical crystallization conditions resulted in two distinct conformations. Although these conformations may be a result of the crystallization process, it supports the idea that GDF8 and GDF11 are flexible enough to be trapped in alternate states. However, an additional apo-GDF11 structure revealed the ligand in the classic “open” conformation and contained similar molecular contacts within the wrist region that we observed in the FS288-bound state [46]. Furthermore, under similar crystallization conditions, additional TGFβ superfamily growth factors have been solved in their apo states and adopt the classic “open” conformation (reviewed in [38]). For example, structures of several BMP ligands in the apo state all exhibit the “open” configuration [77–81]. The extreme “closed” conformation of GDF8 is not entirely unprecedented, as one structure of activin A in complex with ActRIIB showed a similar “closed” configuration [62]. This was initially somewhat controversial; however, multiple activin A structures have since been determined and support the notion that the activin A dimer is flexible [82, 83]. It should be noted that this is not the first example of GDF8 captured in an alternate conformation. Recently, the co-crystal structure of GDF8 bound to a neutralizing antibody was solved where the GDF8 ligand adopts an exaggerated “open” conformation [61], resembling the apo-GDF11 structure presented here. Furthermore, similar to activin A and GDF8, ligands of the TGFβ subclass also exhibit significant flexibility [60, 84–86]. In fact, the biological activity of different TGFβ isoforms, which also share high overall sequence identity, was shown to correlate with the rigidity of the dimer, specifically the wrist helix [60]. However, we cannot discount that the crystallization process has trapped these ligands in alternate conformations. Additional solution-based biochemical approaches are needed to better understand ligand dynamics. Nevertheless, differences in the wrist region of GDF11 could contribute to the increased ligand potency over GDF8 by either stabilizing the dimer interface or presentation of the wrist/pre-helix loop to facilitate differential direct interaction with the type I receptors, or a combination thereof.

Our data strongly indicate that GDF11 is a more potent signaling ligand compared to GDF8. While this observation has not been explicitly realized in the literature, there is evidence suggesting that our results are consistent in that GDF11 is a more potent ligand than GDF8 [16, 44]. However, a recent study utilizing a cell line with modified and potentially functionally inactivated type II and type I receptors showed that GDF8 and GDF11 are nearly indistinguishable in terms of their potential for type II and type I heterodimerization [87]. In this same study and using another assay, there was little difference in the potentiation of downstream SMAD2/3 responsive elements between GDF8 and GDF11 [87]. Interestingly, we observed that differences in SMAD2/3 responsiveness between GDF8 and GDF11 were less pronounced in the HepG2 cell line, the same cell line utilized in [87]. We observed more robust differences between GDF8 and GDF11 in other cell lines and in vivo. Therefore, a potential explanation for these differences may be due to the receptor profile of a particular cell type or tissue in addition to other confounding factors such as co-receptors. Given that GDF8 may not utilize the type I receptors as effectively as GDF11, it is tempting to speculate that both potency and receptor utilization differentiate the biological actions of GDF11 and GDF8.

## Conclusions

In conclusion, we present evidence supporting the notion that GDF8 and GDF11, despite their high sequence identity, are not functionally equivalent. Our data show that GDF11 is a more potent activator of SMAD2/3 in vitro and in vivo. GDF8 has been considered a somewhat unique ligand of the TGFβ family due to dual utilization of the type I receptors ALK4 and ALK5. Our data suggest that GDF11 possesses these same attributes but, due to differences in amino acid composition, utilizes these receptors more effectively to initiate signal transduction. Apart from additional regulatory alterations between GDF8 and GDF11, these ligands may have evolved differences in relative potency through selective pressure in order to provide an evolutionary advantage.

## Methods

### Purified proteins utilized in this study

Proteins used in this study were commercially purchased or produced and purified as previously described with minor alteration [47, 48, 50, 52–55, 88, 89]. Unless otherwise noted, experiments were performed with proteins produced and purified by the authors. Purchased proteins were as follows: mature GDF8 (R&D Systems; Cat. no. 788-G8-CF and PeproTech; Cat. no. 120-00), mature GDF11 (R&D Systems; Cat. no. 1958-GD-010-CF and PeproTech (Cat. no. 120-11); Fc-ALK5 (R&D Systems; Cat. no. 3025-BR/CF), Fc-ActRIIB (R&D Systems; Cat. no. 339-RB/CF). Mature ligands purchased from R&D Systems were reconstituted in 4 mM HCl, 0.01% bovine serum albumin (BSA). Mature ligands purchased from PeproTech utilized in the in vitro studies were reconstituted in 4 mM HCl, 0.01% BSA. Produced and purified proteins are as follows: mature GDF8 [6, 18, 47, 50, 54, 89], mature GDF11 [89], latent GDF8/GDF11 chimeras (see below), mature activin A [52, 55, 62], mature activin B (see below), mature TGFβ3 [73], TβRII [73], FS288 [52, 54], FSTL3 [47, 55], GASP1 and GASP2 [50, 90], ActRIIB-ECD [62], and ALK5-ECD [73].

#### Activin B

Chinese hamster ovary (CHO)-DG44 cells were co-transfected with linearized plasmids containing full-length human activin B (pcDNA3) and dihydrofolate reductase (pMT2). The transfected cells underwent multiple rounds of clonal selection performed with increasing concentrations of up to 1.24 μM methotrexate (MTX) to generate a stable cell line expressing activin B. High expressing clones were isolated and adapted to suspension culture in SFM4CHO-Utility (HyClone). Activin B was produced by adjusting the cells at 1 × 10^6^ cells/mL to 5 mM sodium butyrate and culturing for 8 days, after which the conditioned medium was concentrated and purified as previously described [91].

#### GDF8/GDF11 chimeras

GDF11-like changes were introduced into a pRK5 plasmid containing wild-type full-length human GDF8 using site-directed mutagenesis. GDF8/GDF11 chimeric protein was transiently produced using HEK293T co-transfected with human furin in pcDNA4 using polyethylenimine MAX (Polysciences, Inc.). Transfection proceeded for 4 h in culture medium followed by exchange into serum-free media. Conditioned media were collected 72–96 h later and concentrated 10 times. The concentrated media were applied to a Superdex S200 column (Amersham Biosciences). Fractions containing the latent complex were pooled and concentrated. Initial protein concentration and subsequent normalization were done by quantification (ImageJ) of SDS-PAGE colloidal Coomassie-stained gels.

### GDF11:FS288 complex crystal structure determination

Purified GDF11 (Acceleron Pharma) was mixed with FS288 at a 1:2.5 molar ratio and purified over a Superdex 200 column (Amersham Biosciences) similar to previous purification of the GDF8:FS288 complex [54]. The protein was then concentrated to 6.6 mg/mL and mixed 1:1 in a hanging drop experiment with a solution containing 125 mM phosphate citrate pH 4.2, 18% (w/v) EtOH, and 1% (w/v) PEG 1000. Diffraction experiments were performed at the Argonne National Laboratory Advanced Photon Source 23ID beamline and processed as previously described [54]. Phasing was performed by molecular replacement using Phaser and the CCP4 suite using one dimer of GDF8 and one monomer of FS288 as the search model (PDB ID: 3HH2; [54]). Refinement was carried out with Refmac and Phenix. Coordinates have been deposited in the PDB (PDB ID: 5JHW).

### apo-GDF8 crystal structure determination

Crystals of apo-GDF8 were obtained while attempting to generate crystals of the GDF8:GASP1 complex. Purified mature GDF8 [50, 54] was mixed at a 1:1.5 molar ratio with purified GASP1 [50, 90], and the complex was purified as previously described [50]. The complex was concentrated to 10 mg/mL and mixed 1:1 in a hanging drop experiment with a well solution containing 100 mM MES pH 6.0 and 40% (w/v) 2-methyl-2,4-pentanediol (MPD). Crystals were readily obtained in the presence of noticeable protein precipitant. The resultant crystals contained only GDF8. Diffraction experiments were performed at the Argonne National Laboratory Advanced Photon Source 21ID beamline. Phasing was performed by molecular replacement using MolRep and the CCP4 suite using one monomer of GDF8 as the search model (PDB ID: 3HH2; [54]). Refinement was carried out with Refmac and Phenix. Coordinates have been deposited in the PDB (PDB ID: 5JI1).

### apo-GDF11 crystal structure determination

Crystals of apo-GDF11 were obtained while attempting to generate crystals of the GDF11:ActRIIB complex. Purified mature GDF11, provided by Acceleron Pharma, was mixed at a 1:2.1 ratio with purified ActRIIB-ECD, and the complex was sized using a Superdex S75 column (Amersham Biosciences). The complex was concentrated to 7.5 mg/mL and mixed 1:1 in a hanging drop experiment with a well solution containing 5 mM CaCl_2,_ 0.1 M sodium acetate pH 3.75, and 33% MPD. Similar to apo-GDF8, crystals were readily obtained in the presence of noticeable precipitant. The resultant crystals contained only GDF11. Diffraction experiments were performed at the Argonne National Laboratory Advanced Light Source 23-ID-D beamline. Phasing was performed by molecular replacement using Phaser and the CCP4 suite using one monomer of GDF11 as the search model (PDB ID: 5E4G). Refinement was carried out with Refmac and Phenix. Coordinates have been deposited in the PDB (PDB ID: 5UHM).

### Luciferase reporter assays

#### HEK293 activity/potency and inhibition assays

The assays using the HEK293-(CAGA)_12_ luciferase reporter cells (initially derived from RRID: CVCL_0045) were performed as previously described [47–50]. Cells were seeded in a 96-well plate and grown for 24 h. For the activity comparison assays, the growth medium was then removed and replaced with serum-free medium + 0.1% BSA containing a twofold serial dilution series of mature GDF8 or GDF11 for 18 h. Inhibition assays were performed in a similar fashion, except that the ligand was held at a final concentration of 0.62 nM and then mixed with twofold serial dilutions of antagonist (FS288, FSTL3, GASP1, GASP2, ActRIIB-ECD, Fc-ActRIIB; R&D Systems). The cells were lysed and luminescence was recorded immediately using a Synergy H1 Hybrid plate reader (BioTek). The activity data were imported into GraphPad Prism and fit using a non-linear regression with a variable slope to calculate the EC_50_ or IC_50_.

#### HEK293-GDF8/GDF11 chimeras

For these transfection assays, the HEK293-(CAGA)_12_ luciferase reporter cells were plated in a 96-well plate at ~2 × 10^4^ cells/well, grown for 24 h, and transiently co-transfected with plasmids containing GDF8/GDF11 chimeric constructs (25–100 ng; pRK5), human TLL2 (50 ng; pcDNA3), and human furin (50 ng; pcDNA4) using Mirus LT-1 transfection reagent. Empty pRK5 vector was added for a total of 200 ng DNA transfected/well. Transfection proceeded overnight followed by removal of growth medium in exchange for serum-free medium. Cells were cultured for an additional 24 h and assessed for luciferase activity as described above. The cells were plated and cultured similarly for the experiments where exogenous complexes were added to the cells. Following acid activation with 1 M HCl, the complexes were neutralized with 1 M NaOH and diluted in serum-free medium + 0.1% BSA. The culture medium was removed and the media containing the complexes was added to the cells. After 18–24 h, the cells were lysed and assessed for luciferase activity. For the TLL2 activation experiments, the cells were transfected with 50 ng TLL2 24 h prior to treatment with complexes diluted in serum-free media + 0.01% BSA. Cells were lysed 18–24 h later and assessed for luciferase activity.

#### HepG2 and LβT2 activity assays

The luciferase and hormone assays in HepG2 (ATCC; Cat. no. HB-8065; RRID: CVCL_0027) and LβT2 (kindly provided by Dr. Pamela Mellon, UCSD; RRID: CVCL_0398) cells were performed as previously described [51, 92]. Ligands were purchased from commercial sources for these experiments.

#### RIB L17 receptor utilization assays

The assays using RIB L17 (kindly provided by Dr. Joan Massagué; RRID: CVCL_0596) cells were performed as previously described with some minor alterations [54]. To increase the experiment scale, the assay was performed in a 96-well plate. The cells were plated at ~2 × 10^4^ cells/well and grown for 24 h. The cells were then co-transfected with a total of 100 ng DNA containing the (CAGA)_12_ luciferase reporter construct (gift from Dr. Anita Roberts [93]) and receptor containing plasmids (pRK5 rat ALK4, pRK5 rat ALK5, pcDNA3 human ALK7) alone or in combination using Mirus LT-1 transfection reagent. The ALK7 S270T variant was produced via mutagenesis with the following primer set: forward, GACTCAACTTTGGCTGGTAACTGAATATCATGAACAGGG; reverse, CCCTGTTCATGATATTCAGTTACCAGCCAAAGTTGAGTC. Empty pRK5 vector was added to normalize the total DNA concentration. Transfection proceeded overnight in culture medium followed by media exchange to serum-free media + 0.1% BSA containing 0.62 nM mature activin A, activin B, GDF8, GDF11, or TGFβ3. After 8 h, the cells were lysed and assessed for activity.

#### Surface plasmon resonance (SPR) studies

SPR analysis was performed similarly to previous studies [47, 50]. Briefly, experiments and protein dilutions were carried out in HBS-EP+ buffer (10 mM HEPES, pH 7.4, 500 mM NaCl, 3.4 mM EDTA, 0.05% P-20 surfactant, 0.5 mg/mL BSA) at 37 °C on a Biacore T200 optical biosensor system. All experiments were performed using a CM5 biosensor chip. Proteins were either immobilized by standard amine chemistry according to the manufacturer’s protocol or captured by using immobilized Protein A. For the specific experimental design, see the Results section. Data were analyzed using BIAevaluation software version 1.0.

### Heparin affinity analysis

Determination of heparin affinity was performed as previously described [54]. Briefly, 100 μg of FS288 alone or in complex with GDF8 or GDF11 was applied to a 1 mL HiTrap column (Amersham Biosciences) and eluted with a linear 2 M NaCl gradient over 120 column volumes.

### Primary skeletal myoblasts

Primary skeletal myoblasts were isolated from limb muscles of mice aged 8–12 weeks. After dissection, limb muscles were washed in ice-cold phosphate-buffered saline (PBS). Next, tissues were removed from PBS, minced with surgical scissors, and placed in digestion media (0.2% collagenase type II, 0.05% dispase in DMEM) for 15 min while shaking at 37 °C. After digestion, muscle slurries were triturated until smooth and then digested for additional 8 min. 5 mL filtered donor bovine serum was added to stop the enzymatic reaction and the digestion, and the mixture was triturated again until smooth followed by addition of PBS and centrifuged at 1600 rpm for 5 min. Pellets were resuspended in PBS and filtered through a 70-μm cell strainer and centrifuged at 1600 rpm for 5 min. Pellets were resuspended in growth media (20% horse serum, 1% Glutamax, 1% penicillin/streptomycin, 5 ng/mL bFGF (Sigma-Aldrich) in F10 media) and added to coated culture dishes (0.2% rat-tail collagen, 5 μg/mL natural mouse laminin). Media were replaced after 48 h of culture. Cells were grown for another 48 h before the culture was enriched for myoblasts. Detached myoblasts were collected and centrifuged at 1200 rpm for 5 min and then re-plated on freshly coated culture dishes. Purity of the myoblast culture was assessed microscopically, and a second round of pre-plating was performed to maximize the purity before treating primary myoblasts with commercially purchased GDF8 or GDF11 (PeproTech; Cat. no. 120-00 and Cat. no. 120-11, respectively). Western analysis on cell lysates was performed using phosphorylated SMAD2/3 (Cell signaling; Cat. no. 8828S; Lot 6), total SMAD2/3 (Cell signaling; Cat. no. 3102S; Lot 9), and GAPDH (Santa Cruz; Cat. no. sc-25778; Lot I3015) primary antibodies. Antibody detection was performed with horseradish peroxidase-conjugated antibodies (Cell signaling; Cat. no. 7074S; Lot 25) and enhanced chemiluminescence (Amersham™ GE Healthcare; Cat. no. 45-002-401).

### In vivo injection of ligands into mice

All animal studies were performed as approved by the Harvard Committee on Animals. Adult (1-year-old) C57Bl/6 male mice were obtained from Charles River, and intravenously injected (by tail vein injection) with 0.5 mg/kg GDF11 (PeproTech) or 0.5 mg/kg, 1 mg/kg, 2 mg/kg and 4 mg/kg GDF8 (R&D Systems) or saline as control. Ligands were reconstituted in water at a concentration of 1 mg/mL and diluted in saline prior to injection. Heart tissue was collected 1 h post injection. Whole heart protein lysates were obtained by homogenizing the heart in RIPA buffer freshly supplemented with 1 mM PMSF and protein phosphatase inhibitor 2 and 3 (Sigma-Aldrich). 40 g total protein was loaded in NuPAGE 4-12% Bis-Tris gels (LifeTechnologies). Following transfer, membranes were blocked with non-fat dry milk for 1 h at room temperature and successively incubated with primary pSMAD2 antibody (Millipore, Cat. no. AB3849; Lot 2649232) and total SMAD2/3 antibody (Cell Signaling Technology, Cat. no. 8685P; Lot 4) overnight at 4 °C. Proteins were detected with horseradish peroxidase-conjugated antibodies (BioRad Laboratories; Cat. no. 172-1019; Lot L006328 A) and enhanced chemiluminescence (Amersham™ GE Healthcare, Cat. no. RPN2236).

## Additional files

Additional file 1: Table S1. Analysis of GDF8 and GDF11 activity in HEK293, HepG2 and LβT2 cells [57, 94–96]. (DOC 30 kb)
Additional file 2: Figure S1. Potency of recombinant GDF8 and GDF11 from different sources. Luciferase reporter gene assay ((CAGA)_12_ promoter) following titration of GDF8 (*blue*) and GDF11 (*orange*) ligands in HEK293 cells. Luciferase activity was assessed 18–24 h post ligand treatment. The calculated EC50 value for each ligand source using non-linear regression with variable slope is shown in the *table* below the graph. Data information: Data are presented as percent GDF11 activation after background subtraction (0 nM ligand concentration). Each *point* is the mean ± SEM of three to four independent experiments. Ligand sources are indicated in the graph. (TIF 750 kb)
Additional file 3: Figure S2. Pulse-chase treatment with GDF8 or GDF11 reveals potency differences. A Short exposure to GDF11 results in a significantly enhanced SMAD3-dependent response compared to GDF8. The experimental design is such that the ligand was added to HEK293 cells stably transfected with the (CAGA)_12_ promoter driving the luciferase gene for the indicated time followed by replacement of media without ligand. Activity was measured 24 h after initial treatment. Cells were treated with GDF8 or GDF11 at a ligand concentration of 620 pM. B Time-dependent differences in the SMAD3 activation by GDF8 and GDF11. Similar experimental design as in B, but instead cells were lysed and assessed for luciferase activity at the indicated time of ligand treatment. Cells were treated with GDF8 or GDF11 at a ligand concentration of 620 pM. Data information: Data are presented as fold activation above background (0 nM ligand concentration). Each *point* is the mean ± SEM of three independent experiments. Curves were compared using two-way ANOVA with Bonferroni correction (****P* ≤ 0.001). Ligand sources: GDF8 and GDF11 obtained from Acceleron Pharma. (TIF 256 kb)
Additional file 4: Figure S3. Sequence alignment of human BMP2, GDF8, and GDF11. *Gray bars* above and below the sequence depict gross topology of the ligands. Residues that interact with the type I receptor (*blue*) and type II receptor (*yellow*) are shown on BMP2 based on the BMP2:ALK3:ActRIIA co-crystal structure (Protein Data Bank (PDB): 2GOO; [97]). The non-identical residues between GDF8 and GDF11 are highlighted in *gray*. (TIF 566 kb)
Additional file 5: Figure S4. Binding of GDF11 to the type I receptor ALK5. A, B, C Steady state analysis for SPR traces shown in Fig. 7b and calculated values. The maximum response at each concentration is plotted to a steady state binding equation using Biacore T200 Evaluation Software version 1.0 (Biacore). Sensorgrams were double referenced using an average of two 0 nM ligand injections. Ligand sources: GDF8 and GDF11, gift from Acceleron Pharma; Activin A, Activin B, and TGFβ3, produced and purified as described in “Methods.” D, E, F Ligand binding to Fc-ActRIIB-ECD (A), Fc-ALK5-ECD (B), and ALK5-ECD (C) amine coupled to a CM5 biosensor chip. Ligands were at 500 nM. TβRII, the type II receptor, was required for TGFβ3 binding to Fc-ALK5-ECD and ALK5-ECD. The receptor concentration was at 1 μM for this experiment. Experiments were performed using 40 μL/min flow rate at 37 °C. (TIF 1166 kb)
Additional file 6: Figure S5. Purification and quantification of GDF8/GDF11 chimeric ligands. A, B Representation of purified protein from selected GDF8/GDF11 chimeras under non-reduced (A) and reduced (B) conditions (4–15% gradient gel). Chimeras or empty vector control were produced transiently using HEK293T cells and purified using size exclusion chromatography. The resultant peak containing the prodomain:mature ligand complex was pooled and concentrated. For empty vector control, corresponding fractions from a similar retention volume were pooled. The lane labeled “pro domain + mature” serves as a control for the molecular weight of purified wt GDF8 prodomain and wt GDF8 mature ligand. Note the expected changes in mass of the mature ligand (*blue arrows*) under non-reducing (dimer) and reducing (monomer) conditions while the prodomain mass is relatively unaffected (*gray arrow*). Protein is visualized by colloidal Coomassie stain. To ensure that comparable amounts of each GDF8/GDF11 chimeric protein were being administered in the cell-based assays, we first normalized protein concentrations based on the amount of dimer present in a non-reduced SDS-PAGE gel stained with colloidal Coomassie. The samples were then normalized and reexamined by SDS-PAGE gel under non-reducing and reducing gel. The subsequent bands were quantified (*bottom, below gel*) under non-reduced (A) and reduced (B) gels using ImageJ showing that the protein concentrations were indeed normalized. 500 ng of recombinant GDF8 prodomain and purified GDF8 mature were loaded for reference. (TIF 3694 kb)
