# Supplementary material for: Structural basis for potency differences between GDF8 and GDF11
Source: BMC Biol. 2017 Mar 3;15:19. doi: 10.1186/s12915-017-0350-1 (PMC5336696; doi:10.1186/s12915-017-0350-1)
Supplement: Additional file 1: Table S1. — Analysis of GDF8 and GDF11 activity in HEK293, HepG2 and LβT2 cells [57, 94–96]. (DOC 30 kb) [file 12915_2017_350_MOESM1_ESM.doc]

**Supplemental Table 1 - Analysis of GDF8 and GDF11 activity in HEK293, HepG2 and Lβ**T2 cells

| Cell type | Ligand | | EC50 (nM) | EC50 (nM) 95% CIa | Max. Fold response | Type I receptor profile | Type II receptor profile |
| --- | --- | --- | --- | --- | --- | --- | --- |
| HEK293 | GDF8 | | 0.48 | 0.46 to 0.50 | 385 | ALK4, ALK5, and ALK7b | ActRIIA and ActRIIBb |
| GDF11 | | 0.08 | | 0.07 to 0.09 | 439 | | |
| HepG2 | GDF8 | | 5.4 | 4.9 to 6.1 | 12 | ALK4, ALK5, and ALK7*b* [51] | ActRIIA and ActRIIB*b* [51] |
| GDF11 | | 3.4 | | 3.1 to 3.7 | 50 | | |
| LβT2 | GDF8 | | 0.08 | 0.067 to 0.098 | 12 | ALK4 [95], ALK5 [96], ALK7 [95] | ActRIIA and ActRIIB [97] |
| GDF11 | | 0.03 | | 0.030 to 0.038 | 14 | | |
