# Supplementary figures and images for: Structural basis for potency differences between GDF8 and GDF11
Source: BMC Biol. 2017 Mar 3;15:19. doi: 10.1186/s12915-017-0350-1 (PMC5336696; doi:10.1186/s12915-017-0350-1)

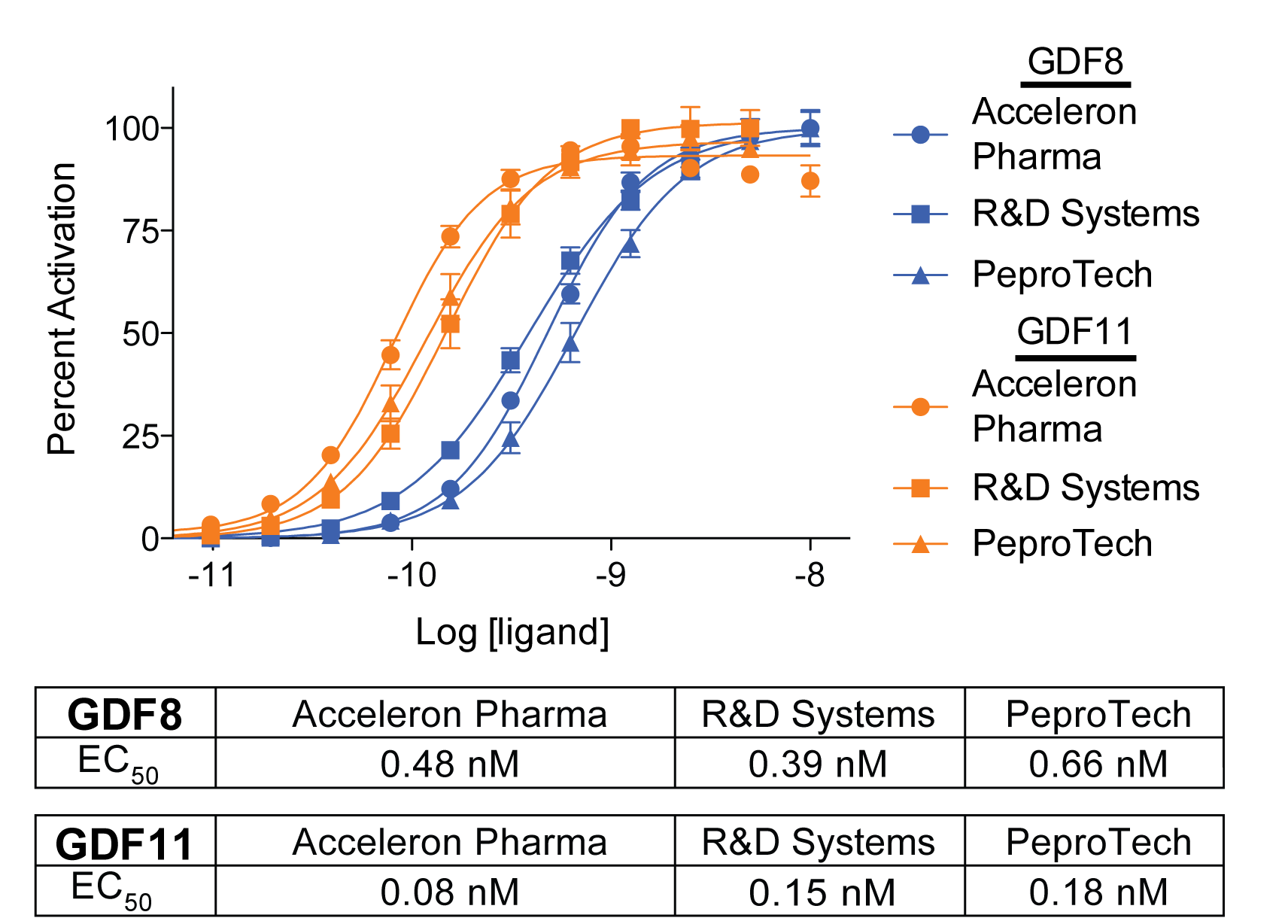

Supplement: Additional file 2: Figure S1. — Potency of recombinant GDF8 and GDF11 from different sources. Luciferase reporter gene assay ((CAGA)12 promoter) following titration of GDF8 (blue) and GDF11 (orange) ligands in HEK293 cells. Luciferase activity was assessed 18–24 h post ligand treatment. The calculated EC50 value for each ligand source using non-linear regression with variable slope is shown in the table below the graph. Data information: Data are presented as percent GDF11 activation after background subtraction (0 nM ligand concentration). Each point is the mean ± SEM of three to four independent experiments. Ligand sources are indicated in the graph. (TIF 750 kb) [file 12915_2017_350_MOESM2_ESM.tif]

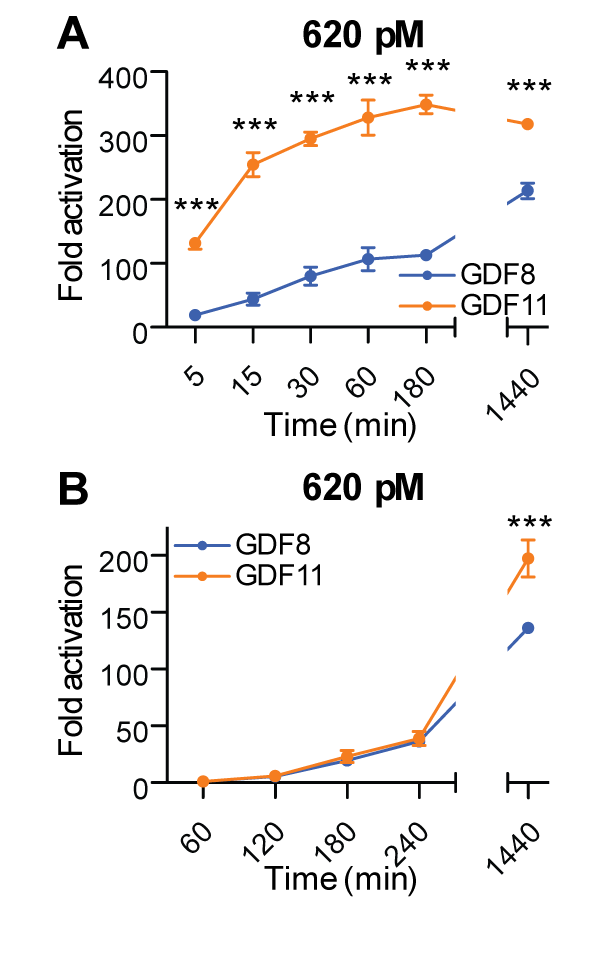

Supplement: Additional file 3: Figure S2. — Pulse-chase treatment with GDF8 or GDF11 reveals potency differences. A Short exposure to GDF11 results in a significantly enhanced SMAD3-dependent response compared to GDF8. The experimental design is such that the ligand was added to HEK293 cells stably transfected with the (CAGA)12 promoter driving the luciferase gene for the indicated time followed by replacement of media without ligand. Activity was measured 24 h after initial treatment. Cells were treated with GDF8 or GDF11 at a ligand concentration of 620 pM. B Time-dependent differences in the SMAD3 activation by GDF8 and GDF11. Similar experimental design as in B, but instead cells were lysed and assessed for luciferase activity at the indicated time of ligand treatment. Cells were treated with GDF8 or GDF11 at a ligand concentration of 620 pM. Data information: Data are presented as fold activation above background (0 nM ligand concentration). Each point is the mean ± SEM of three independent experiments. Curves were compared using two-way ANOVA with Bonferroni correction (***P ≤ 0.001). Ligand sources: GDF8 and GDF11 obtained from Acceleron Pharma. (TIF 256 kb) [file 12915_2017_350_MOESM3_ESM.tif]

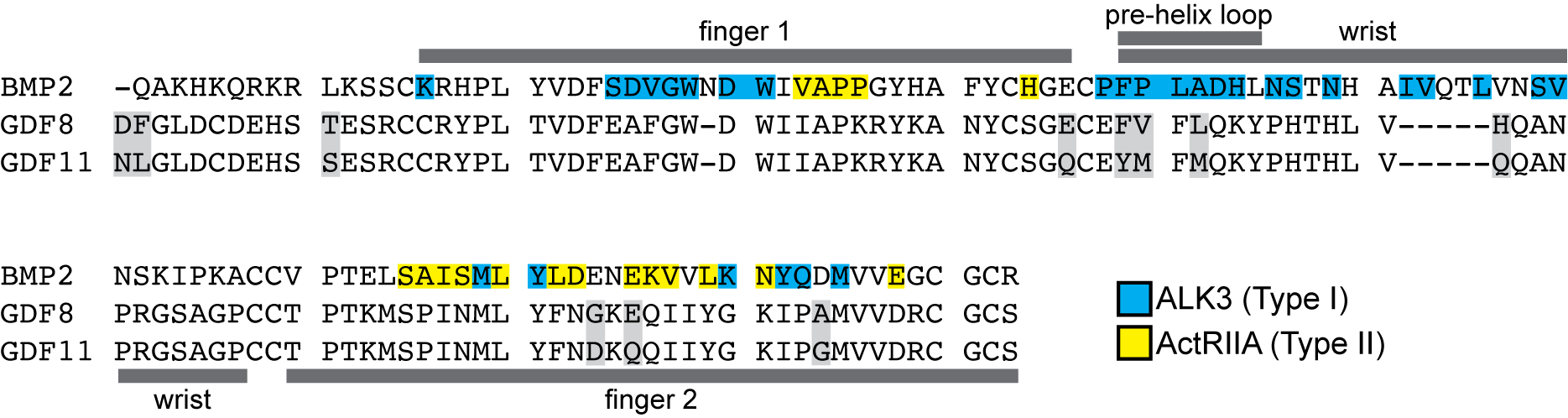

Supplement: Additional file 4: Figure S3. — Sequence alignment of human BMP2, GDF8, and GDF11. Gray bars above and below the sequence depict gross topology of the ligands. Residues that interact with the type I receptor (blue) and type II receptor (yellow) are shown on BMP2 based on the BMP2:ALK3:ActRIIA co-crystal structure (Protein Data Bank (PDB): 2GOO; [97]). The non-identical residues between GDF8 and GDF11 are highlighted in gray. (TIF 566 kb) [file 12915_2017_350_MOESM4_ESM.tif]

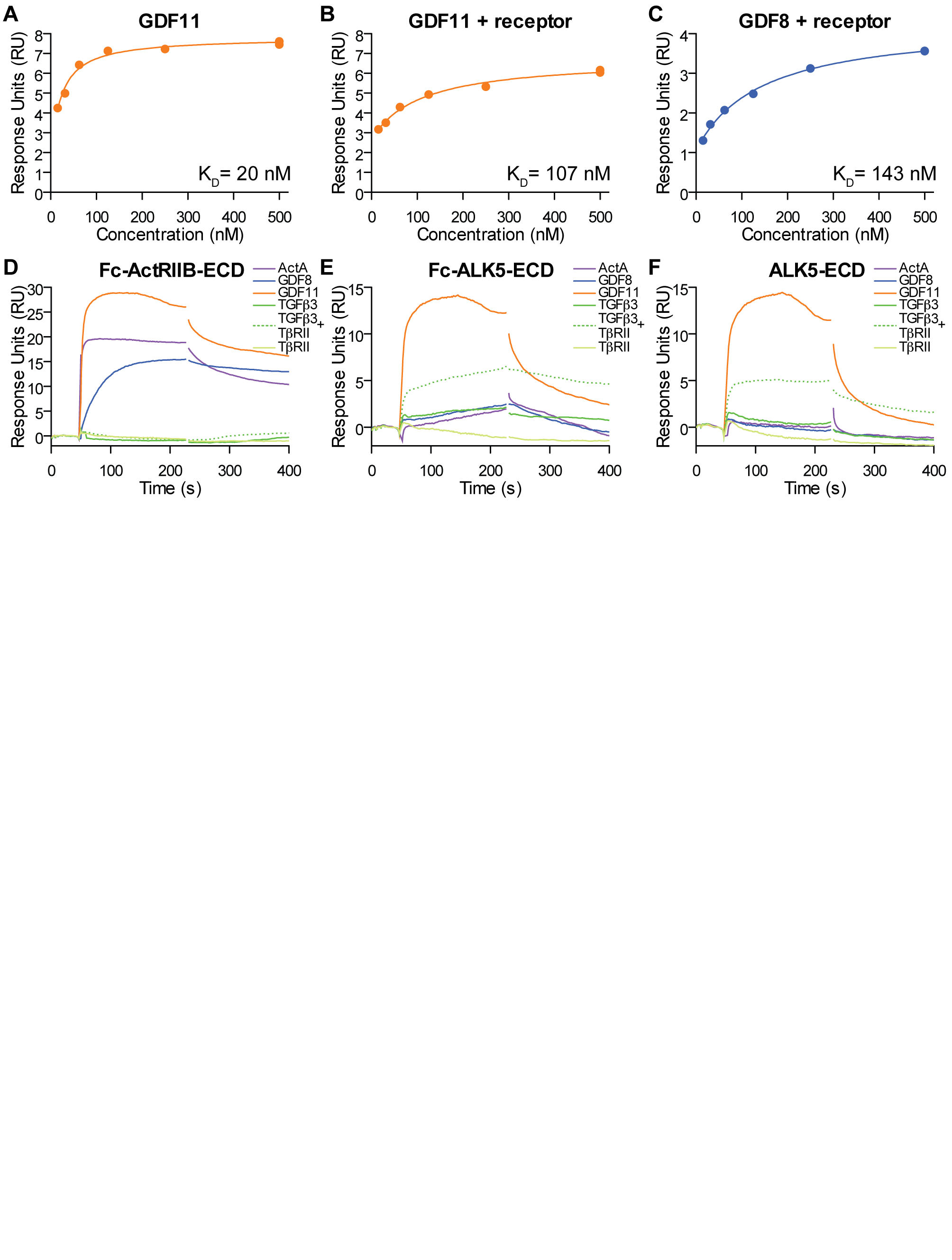

Supplement: Additional file 5: Figure S4. — Binding of GDF11 to the type I receptor ALK5. A, B, C Steady state analysis for SPR traces shown in Fig. 7b and calculated values. The maximum response at each concentration is plotted to a steady state binding equation using Biacore T200 Evaluation Software version 1.0 (Biacore). Sensorgrams were double referenced using an average of two 0 nM ligand injections. Ligand sources: GDF8 and GDF11, gift from Acceleron Pharma; Activin A, Activin B, and TGFβ3, produced and purified as described in “Methods.” D, E, F Ligand binding to Fc-ActRIIB-ECD (A), Fc-ALK5-ECD (B), and ALK5-ECD (C) amine coupled to a CM5 biosensor chip. Ligands were at 500 nM. TβRII, the type II receptor, was required for TGFβ3 binding to Fc-ALK5-ECD and ALK5-ECD. The receptor concentration was at 1 μM for this experiment. Experiments were performed using 40 μL/min flow rate at 37 °C. (TIF 1166 kb) [file 12915_2017_350_MOESM5_ESM.tif]

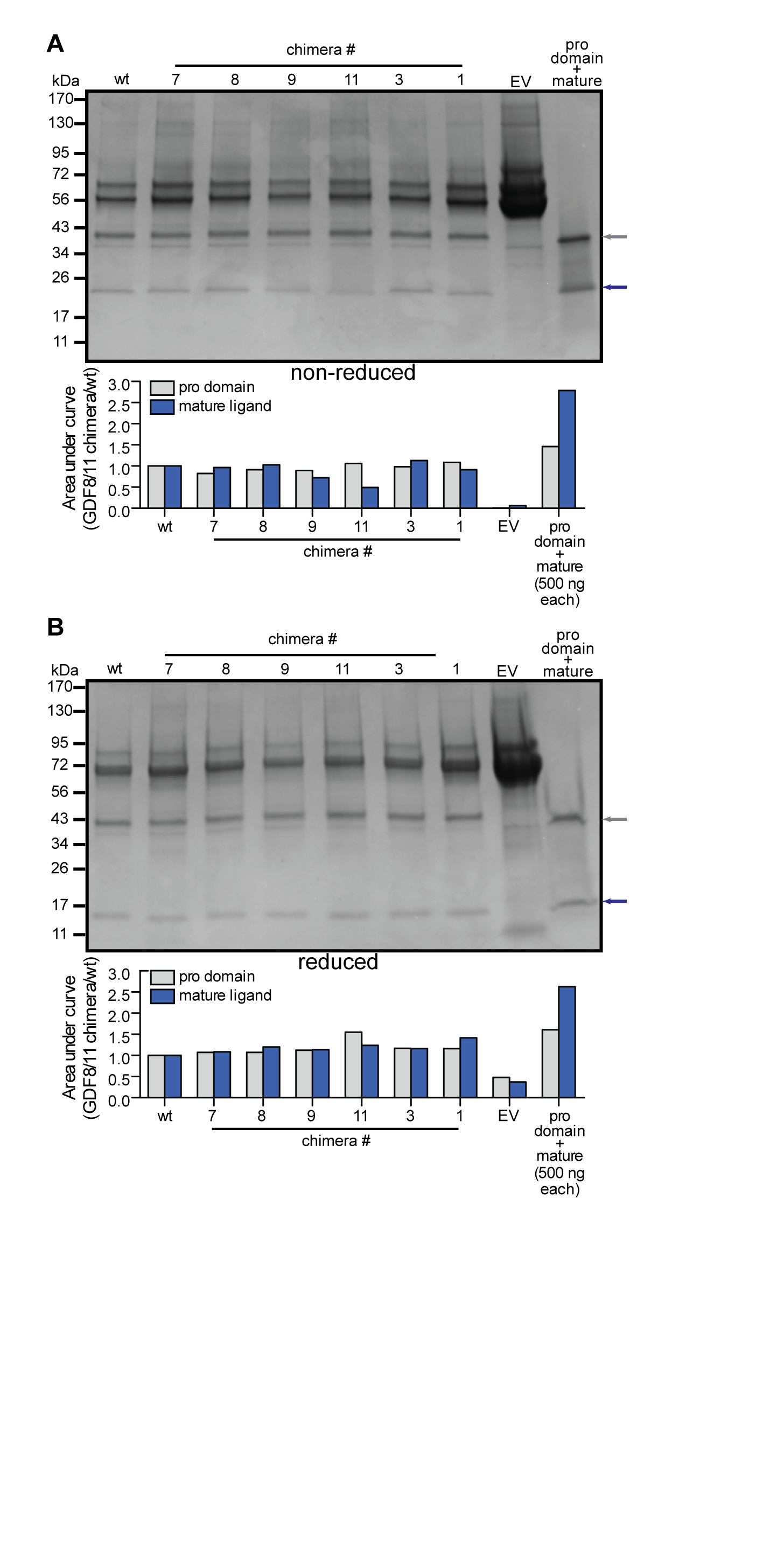

Supplement: Additional file 6: Figure S5. — Purification and quantification of GDF8/GDF11 chimeric ligands. A, B Representation of purified protein from selected GDF8/GDF11 chimeras under non-reduced (A) and reduced (B) conditions (4–15% gradient gel). Chimeras or empty vector control were produced transiently using HEK293T cells and purified using size exclusion chromatography. The resultant peak containing the prodomain:mature ligand complex was pooled and concentrated. For empty vector control, corresponding fractions from a similar retention volume were pooled. The lane labeled “pro domain + mature” serves as a control for the molecular weight of purified wt GDF8 prodomain and wt GDF8 mature ligand. Note the expected changes in mass of the mature ligand (blue arrows) under non-reducing (dimer) and reducing (monomer) conditions while the prodomain mass is relatively unaffected (gray arrow). Protein is visualized by colloidal Coomassie stain. To ensure that comparable amounts of each GDF8/GDF11 chimeric protein were being administered in the cell-based assays, we first normalized protein concentrations based on the amount of dimer present in a non-reduced SDS-PAGE gel stained with colloidal Coomassie. The samples were then normalized and reexamined by SDS-PAGE gel under non-reducing and reducing gel. The subsequent bands were quantified (bottom, below gel) under non-reduced (A) and reduced (B) gels using ImageJ showing that the protein concentrations were indeed normalized. 500 ng of recombinant GDF8 prodomain and purified GDF8 mature were loaded for reference. (TIF 3694 kb) [file 12915_2017_350_MOESM6_ESM.tif]
